# Safety and Immunogenicity of a 4-Component Generalized Modules for Membrane Antigens *Shigella* Vaccine in Healthy European Adults: Randomized, Phase 1/2 Study

**DOI:** 10.1093/infdis/jiae273

**Published:** 2024-06-10

**Authors:** Isabel Leroux-Roels, Cathy Maes, Francesca Mancini, Bart Jacobs, Eleanna Sarakinou, Azhar Alhatemi, Jasper Joye, Silvia Grappi, Giulia Luna Cilio, Alimamy Serry-Bangura, Claudia G Vitali, Pietro Ferruzzi, Elisa Marchetti, Francesca Necchi, Rino Rappuoli, Iris De Ryck, Jochen Auerbach, Anna M Colucci, Omar Rossi, Valentino Conti, Francesco Berlanda Scorza, Ashwani Kumar Arora, Francesca Micoli, Audino Podda, Usman N Nakakana, the Shigella Project Team, Giulia Ranzato, Kishor Mariyala, Sateesh Aravapalli, Stefania Barbucci, Rob Mulder, Francesco Citiulo, Emilia Cappelletti, Gianmarco Gasperini, Carlo Giannelli, Alessandra Acquaviva, Luigi Sollai, Renzo Alfini, Maria Grazia Aruta, Laura Bartle Martin

**Affiliations:** Center for Vaccinology, Ghent University and Ghent University Hospital, Ghent, Belgium; Center for Vaccinology, Ghent University and Ghent University Hospital, Ghent, Belgium; GSK Vaccines Institute for Global Health, GSK, Siena, Italy; Center for Vaccinology, Ghent University and Ghent University Hospital, Ghent, Belgium; GSK Vaccines Institute for Global Health, GSK, Siena, Italy; Center for Vaccinology, Ghent University and Ghent University Hospital, Ghent, Belgium; Center for Vaccinology, Ghent University and Ghent University Hospital, Ghent, Belgium; VisMederi, Siena, Italy; Vaccines Global Safety, GSK, Siena, Italy; Vaccines Global Safety, GSK, Siena, Italy; Vaccines Toxicology Center of Excellence, GSK Siena, Italy; GSK Vaccines Institute for Global Health, GSK, Siena, Italy; GSK Vaccines Institute for Global Health, GSK, Siena, Italy; GSK Vaccines Institute for Global Health, GSK, Siena, Italy; GSK Biologicals, Siena, Italy; Vaccines Global Safety, GSK, Siena, Italy; GSK Vaccines Institute for Global Health, GSK, Siena, Italy; GSK Vaccines Institute for Global Health, GSK, Siena, Italy; GSK Vaccines Institute for Global Health, GSK, Siena, Italy; GSK Vaccines Institute for Global Health, GSK, Siena, Italy; GSK Vaccines Institute for Global Health, GSK, Siena, Italy; GSK Vaccines Institute for Global Health, GSK, Siena, Italy; GSK Vaccines Institute for Global Health, GSK, Siena, Italy; GSK Vaccines Institute for Global Health, GSK, Siena, Italy; GSK Vaccines Institute for Global Health, GSK, Siena, Italy

**Keywords:** altSonflex1-2-3, *Shigella* vaccine, safety, immunogenicity, O-antigen

## Abstract

**Background:**

We report data from stage 1 of an ongoing 2-staged, phase 1/2 randomized clinical trial with a 4-component generalized modules for membrane antigens-based vaccine against *Shigella sonnei* and *Shigella flexneri* 1b, 2a, and 3a (altSonflex1-2-3; GSK).

**Methods:**

Europeans aged 18–50 years (N = 102) were randomized (2:1) to receive 2 injections of altSonflex1-2-3 or placebo at 3- or 6-month interval. Safety and immunogenicity were assessed at prespecified time points.

**Results:**

The most common solicited administration-site event (until 7 days after each injection) and unsolicited adverse event (until 28 days after each injection) were pain (altSonflex1-2-3, 97.1%; placebo, 58.8%) and headache (32.4%; 23.5%), respectively. All serotype-specific functional IgG antibodies peaked 14–28 days after injection 1 and remained substantially higher than prevaccination at 3 or 6 months postvaccination; the second injection did not boost but restored the initial immune response. The highest seroresponse rates (≥4-fold increase in titers over baseline) were obtained against *S. flexneri* 2a (enzyme-linked immunosorbent assay [ELISA] after injection 1, 91.0%; after injection 2 [day 113; day 197], 100%; 97.0% and serum bactericidal activity [SBA] after injection 1, 94.4%; after injection 2, 85.7%; 88.9%) followed by *S. sonnei* (ELISA after injection 1, 77.6%; after injection 2, 84.6%; 78.8% and SBA after injection 1, 83.3%; after injection 2, 71.4%; 88.9%). Immune responses against *S. flexneri* 1b and *S. flexneri* 3a, as measured by both ELISA and SBA, were numerically lower compared to those against *S. sonnei* and *S. flexneri* 2a.

**Conclusions:**

No safety signals or concerns were identified. altSonflex1-2-3 induced functional serotype-specific immune responses, allowing further clinical development in the target population.

***Clinical Trials Registration***. NCT05073003.

## Teaser

In stage 1 of this phase 1/2 study, the 4-component GMMA-based vaccine against *Shigella sonnei* and *Shigella flexneri* 1b, 2a, and 3a (altSonflex1-2-3) induced anti-O-antigen–specific and bactericidal antibodies against all antigens in European adults with no safety signals/concerns identified.

## Graphical abstract

**Graphical abstract:**
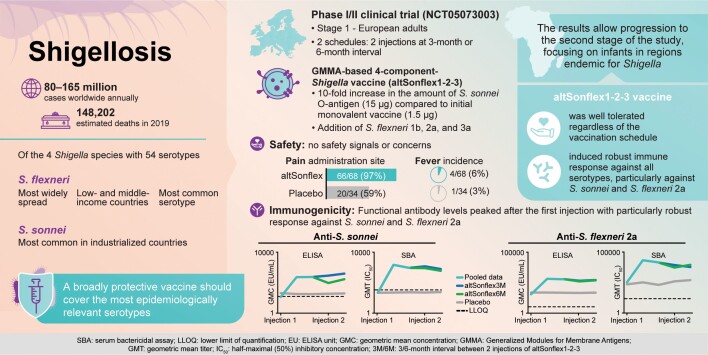

*Shigella* spp. are gram-negative bacteria responsible for shigellosis, an intestinal infection causing severe diarrhea and dysentery that can be life-threatening without adequate care [1, 2]. *Shigella* is one of the leading causes of diarrheal mortality worldwide [3] causing 80–165 million cases annually [4], and an estimated 148 202 deaths in 2019, of which 93 831 occurred in children under 5 years of age [5].

The 54 *Shigella* serotypes cause similar symptoms upon infection, but the immunity is serotype specific [3, 6]. *Shigella flexneri* is widespread in low- and middle-income countries (LMICs), accounting for approximately 60% of infections in infants and children, with the distribution varying widely by location [7]. However, a large proportion of infections are due to *S. flexneri* 2a, with significant contribution of serotypes 1b, 3a, and 6 [8–10]. *Shigella sonnei* is most prevalent in industrialized countries [9, 11]. However, a trend of increasing incidence of *S. sonnei* infections has been observed in LMICs in recent years [12].

Although 2 *Shigella* vaccines are on the market, these are only available in Russia (Shigellvak, Allergen) and China (FS; Lanzhou Institute of Biological Products) [13]. However, several candidate vaccines are in preclinical or clinical development [9, 13, 14]. The O-antigen (OAg) of the bacterial lipopolysaccharide (LPS) is a key target for *Shigella* vaccines as it induces serotype-specific, protective antibodies following natural infection [9].

Generalized modules for membrane antigens (GMMA) provide a promising platform to develop an effective and low-cost vaccine against *Shigella*, which can be readily produced and/or deployed in LMICs [15–18]. A monovalent GMMA-based vaccine against *S. sonnei* (1790GAHB; GSK), assessed in phase 1 and 2 studies, had an acceptable safety profile and elicited bactericidal anti-LPS specific IgG response [19–23], but failed to reach efficacy targets in a phase 2b, randomized, controlled human infection model study [24]. Thus, a new *S. sonnei* construct, with 10-fold higher OAg amount per total GMMA protein was developed and combined with *S. flexneri* serotypes 1b, 2a, and 3a in a new-generation 4-component vaccine candidate (altSonflex1-2-3; GSK), with 15 μg of OAg per serotype [25]. This vaccine composition was chosen according to the most prevalent serotypes and preclinical cross-reactivity data [7, 11].

altSonflex1-2-3 is currently being assessed in a 2-staged, phase 1/2 clinical trial. Here, we report the safety and immunogenicity results of stage 1 in healthy European adults.

## METHODS

### Study Design

This phase 1/2 observer-blind, controlled, randomized, multicountry, age–de-escalation study was conducted in 2 stages: stage 1 (phase 1; first-time in human) in healthy European adults and stage 2 (phase 2) in healthy African adults, children, and infants (ClinicalTrials.gov, NCT05073003). The study was approved by the ethics committee of the Ghent University Hospital and conducted as per Good Clinical Practice guidelines.

Participants were randomized 2:1 to receive either 2 injections of the altSonflex1-2-3 vaccine or a placebo. The study interventions were administered either with a 3-month (altSonflex3M and Placebo3M) or a 6-month interval (altSonflex6M and Placebo6M) (Supplementary Figure 1).

### Study Population

Stage 1 study population were healthy, nonpregnant participants aged 18–50 years, without known prior exposure to *Shigella* or to experimental *Shigella* vaccines and who provided written informed consent. A complete list of inclusion and exclusion criteria is presented in Supplementary Material 1.

### Objectives

The primary objective of stage 1 was assessment of safety and reactogenicity of 2 injections of altSonflex1-2-3 vaccine. Secondary and tertiary objectives included characterization of the vaccine-induced humoral immune profile. Details of stage 1 objectives and end points are available in Supplementary Material 2.

### Study Interventions and Administration

altSonflex1-2-3 or placebo were administered intramuscularly. Each injection of altSonflex1-2-3 contained 15 µg of OAg of each serotype (*S. sonnei* and *S. flexneri* serotypes 1b, 2a, and 3a) adsorbed on aluminum hydroxide (Alhydrogel; Croda) and suspended in buffered saline. The placebo contained all components of the vaccine except antigens, that is, aluminum hydroxide suspended in buffered saline.

### Reactogenicity and Safety Assessment

Safety and reactogenicity outcomes were, for each injection: solicited administration-site adverse events (AEs) (pain, redness, swelling), systemic AEs (only fever), and deviations from reference values of laboratory test results reported during the 7 days following injection, and unsolicited AEs reported during the 28 days following injection; serious AEs (SAEs) were recorded during the entire study (Supplementary Figure 1).

Causality assessment of fever, unsolicited AEs, and SAEs was performed by the investigator I. L.-R.

Solicited and unsolicited AEs were graded 1–3 (mild, moderate, severe), and safety laboratory test results were graded 1–4 (mild to potentially life-threatening) according to adapted Food and Drug Administration toxicity grading scale [26] (Supplementary Material 3). Details of reactogenicity and safety assessments are included in Supplementary Material 3.

### Immunogenicity Assessment

Sera were collected from participants before each vaccine injection (day 1 and day 85/day 169), 14 days after the first injection (day 15), and 28 days after each injection (day 29 and day 113/day 197) (Supplementary Figure 1). *Shigella* serotype-specific serum anti-OAg IgG levels were measured using enzyme-linked immunosorbent assay (ELISA) as detailed in Supplementary Material 4. *Shigella* serotype-specific serum bactericidal antibody levels were measured in a subset of 25% of randomly selected participants using the luminescence-based serum bactericidal activity (SBA) assay, as previously reported [27–29]. Results were expressed in serum titers, that is, serum dilution giving 50% inhibition of bacterial growth (IC_50_). *Shigella* strains used in the study are described in Supplementary Material 5.

### Statistical Analyses

Stage 1 aimed to descriptively evaluate safety and immunogenicity profiles of the study vaccine. Approximately 102 participants were planned to be randomized to achieve 61 evaluable participants in the vaccine arm. All statistical analyses were performed using the SAS software (SAS Institute, Inc). Details of the study sets and analyses, as well as the ELISA and SBA readouts, and the analyses performed are included in Supplementary Material 6. Briefly, for both readouts, for each serotype, geometric mean concentrations (GMCs) or geometric mean titers (GMTs) with the 95% confidence intervals (CIs), within-participant geometric mean ratios (GMRs) with 95% CI, and number and percentage of participants with ≥4-fold increase in concentrations/titers compared to baseline (seroresponse rate) were calculated.

Lower limit of quantification (LLOQ) cutoff values of the ELISA and SBA assays are described in Supplementary Material 6. Values below the LLOQ were set to half the LLOQ value for the analysis purpose.

Safety results are reported as pooled data from the 2 altSonflex1-2-3 groups. As the clinical schedule for the 2 altSonflex1-2-3 groups up to 1 month after injection 1 was identical, immunogenicity data for altSonflex3M and altSonflex6M groups until day 29 were pooled. Data for both placebo groups were also pooled.

## RESULTS

### Study Participants

In total, 102 participants were enrolled in stage 1 between 6 October 2021 and 25 November 2021; 68 were randomized to altSonflex1-2-3 groups (34 each in altSonflex3M and 6M groups), and 34 to the placebo group; 11 participants withdrew from the study (Supplementary Figure 2). For 3 participants, the reason was related to coronavirus disease 2019 (COVID-19) and for 1, to other unsolicited AEs (non-COVID-19); reasons for withdrawal in the remaining participants were not linked to AEs. Stage 1 ended on 6 October 2022.

Demographic characteristics were similar between the study groups (Supplementary Table 1). The mean age at first study vaccination was 34.4 years. All participants were white (Caucasian), and 80.4% were women. Demographic characteristics of participants in the per-protocol set are shown in Supplementary Table 2.

### Reactogenicity and Safety

**Figure 1.**
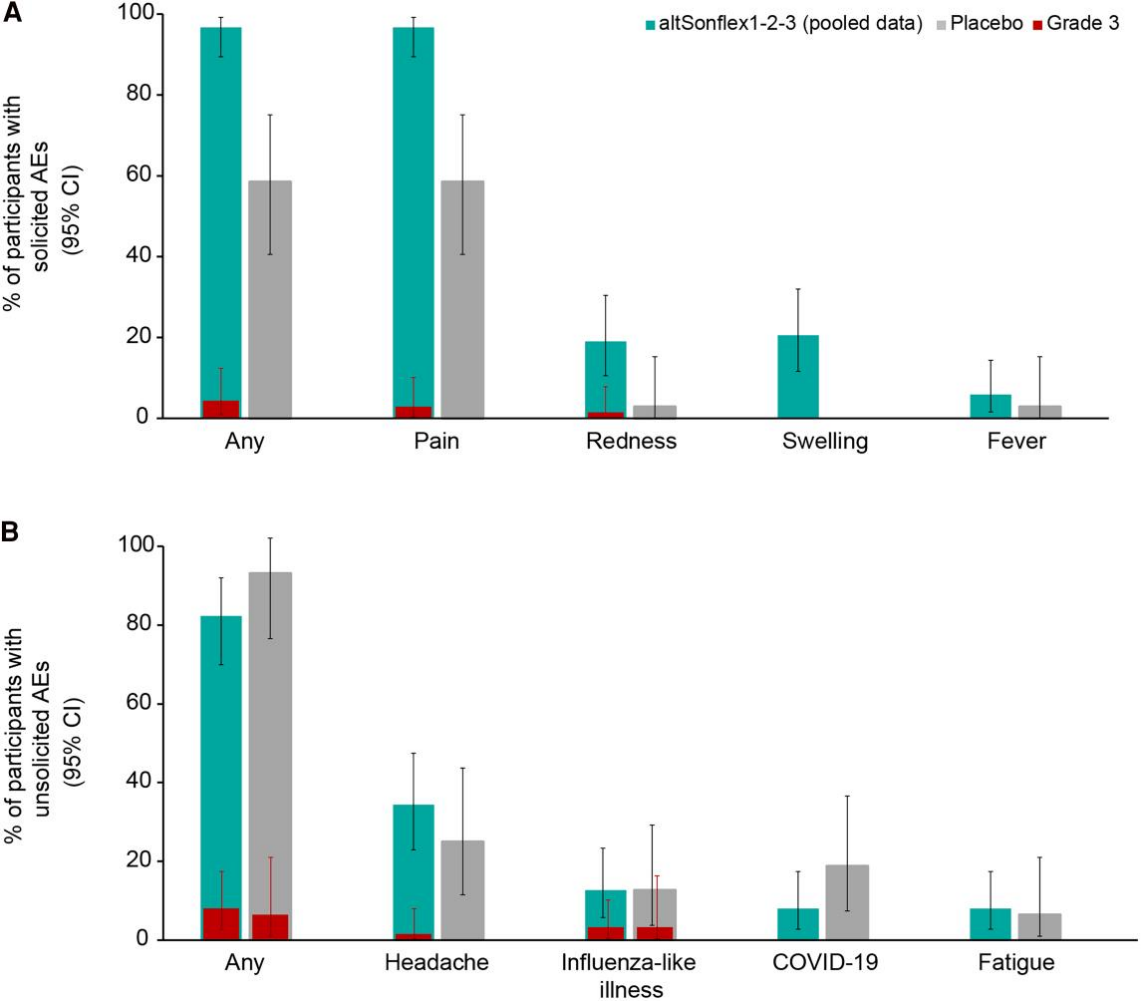
Percentage of participants with (*A*) solicited AEs and (*B*) unsolicited AEs. Abbreviations: AE, adverse event; altSonflex, participants randomized to receive altSonflex1-2-3 vaccine at 3- or 6-month interval (n = 68, pooled data); CI, confidence interval; placebo, participants randomized to receive placebo (n = 34).

The most common solicited administration-site AE was pain, reported by 97.1% and 58.8% of participants in the altSonflex1-2-3 and placebo groups, respectively; grade 3 pain was reported by 2 (2.9%) participants in the altSonflex1-2-3 groups. Fever (≥38°C) was reported by 4 (5.9%) participants from altSonflex1-2-3 groups and 1 (2.9%) participant from the placebo group (Figure 1*A*).

After any injection, at least 1 unsolicited AE was reported in 77.9% and 88.2% of participants in the altSonflex1-2-3 and placebo groups, respectively, the most common being headache (32.4% and 23.5%). These events were all mild-to-moderate in severity except in 1 participant. The most frequent unsolicited AEs were “general disorders and administration site conditions” (42.6%) and “infections and infestations” (29.4%) in the altSonflex 1-2-3 and placebo groups, respectively. Grade 3 unsolicited AEs were reported by 5 (7.4%) and 2 (5.9%) participants in the altSonflex1-2-3 and placebo groups, respectively (Figure 1*B*).

At least 1 unsolicited AE considered causally related to study intervention was reported by 47.1% and 20.6% of participants in the altSonflex1-2-3 and placebo groups, respectively; the most common was headache (23.5% and 8.8%). One participant (altSonflex1-2-3 group) reported 2 grade 3 unsolicited AEs (vomiting and headache) after injection 2 that were considered causally related to the study vaccine. A single SAE (fecaloma), considered unrelated to the study intervention, was reported in 1 participant from the altSonflex1-2-3 group. Safety laboratory analyses did not reveal specific trends or safety signals. Most of the values were within ranges or grade 0 according to the grading scale (data not shown).

### Immunogenicity

#### Shigella sonnei

At baseline, most participants in the altSonflex1-2-3 groups exhibited anti-*S. sonnei* IgG ELISA concentrations below the LLOQ set at 12.8 ELISA units (EU)/mL (Supplementary Material 7). However, some participants in both placebo and altSonflex1-2-3 groups showed detectable baseline concentrations equal or above the LLOQ, with GMCs of 21.0 EU/mL and 11.7 EU/mL, respectively.

**Figure 2.**
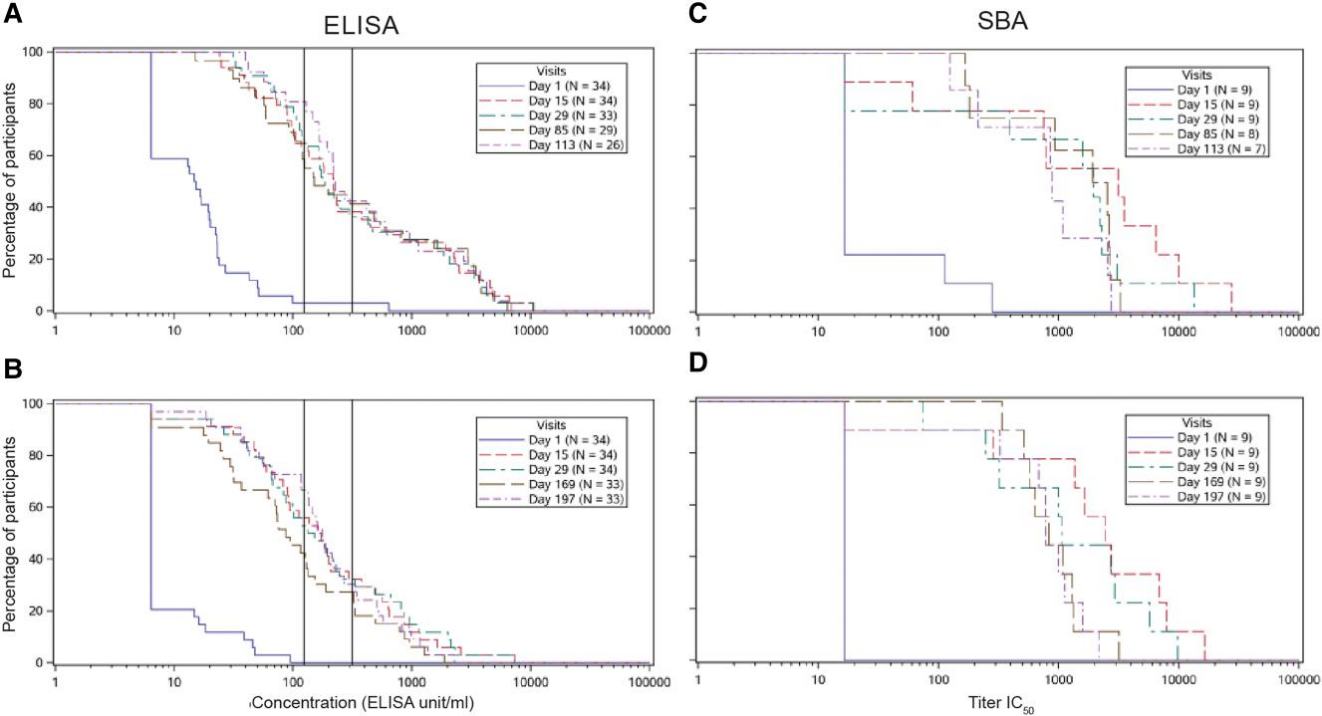
Reverse cumulative distribution curves of (*A* and *B*) anti-*Shigella sonnei* LPS serum IgG as measured by ELISA: (*A*) altSonflex3M group up to 1 month after injection 2; (*B*) altSonflex6M group up to 1 month after injection 2; and (*C* and *D*) bactericidal antibody activity as measured by SBA in a subset of participants: (*C*) altSonflex3M group up to 1 month after injection 2; (*D*) altSonflex6M group up to 1 month after injection 2. Left vertical line shows 1:800 titers in TAU assay, which correspond to 124 EU/mL in the ELISA. Right vertical line shows 1:1600 titers in TAU assay, which correspond to 315 EU/mL in ELISA; see Supplementary Material 6 for further details. Abbreviations: altSonflex3M, participants randomized to receive altSonflex1-2-3 vaccine at 3-month interval; altSonflex6M, participants randomized to receive altSonflex1-2-3 vaccine at 6-month interval; ELISA, enzyme-linked immunosorbent assay; EU, ELISA unit; IC_50_, half maximum inhibitory concentration; IgG, immunoglobulin G; LPS, lipopolysaccharide; n, number of participants with available data at a given time point; SBA, serum bactericidal assay; TAU, Tel Aviv University.

After injection 1, GMCs increased to 229.1 EU/mL at day 15 and 235.5 EU/mL at day 29 in altSonflex1-2-3 groups, while they remained at baseline levels in the placebo group. Before injection 2, GMCs were 287.6 EU/mL and 94.8 EU/mL in altSonflex3M (day 85) and altSonflex6M (day 169), respectively, versus 19.0 EU/mL in the placebo group. The second injection did not boost the initial response, but GMCs reached levels similar to those elicited by the first injection. GMRs (geometric mean of the within-subject ratios of the postvaccination vs baseline) were 20 after injection 1 (pooled altSonflex1-2-3 groups), and 22.8 and 20.1 after injection 2, in altSonflex3M and altSonflex6M, respectively. No increase in GMCs was observed in the placebo group at any time point (Table 1 and Figure 2).

**Table 1.** Immune Response Against *Shigella sonnei* as Measured by ELISA and SBA (Per-Protocol Set)

		Day 1, Baseline		Day 15		Day 29		Day 85^a^/Day 169,^b^ Before Injection 2		Day 113^a^/Day 197^b^	
Parameter	Study Group	n	Value	n	Value or %	n	Value or %	n	Value or %	n	Value or %
ELISA											
GMC, EU/mL	altSonflex3M + altSonflex6M	68	11.7 (9.4–14.6)	68	229.1 (152.5–344.0)	67	235.5 (156.8–353.7)	…	…	…	…
	altSonflex3M	…	…	…	…	…	…	29	287.6 (144.1–574.1)	26	387.8 (206.6–727.8)
	altSonflex6M	…	…	…	…	…	…	33	94.8 (55.2–162.9)	33	167.2 (104.3–267.9)
	Placebo	34	21.0 (13.2–33.6)	32	18.4 (11.6–29.3)	32	18.1 (11.5–28.6)	28	19.0 (11.1–32.3)	28	19.9 (11.8–33.7)
4-fold increase,^c^ %	altSonflex3M + altSonflex6M	…	…	68	76.5 (64.6–85.9)	67	77.6 (65.8–86.9)	…	…	…	…
	altSonflex3M	…	…	…	…	…	…	29	75.9 (56.5–89.7)	26	84.6 (65.1–95.6)
	altSonflex6M	…	…	…	…	…	…	33	63.6 (45.1–79.6)	33	78.8 (61.1–91.0)
	Placebo	34	…	32	0.0 (.0–10.9)	32	0.0 (.0–10.9)	28	0.0 (.0–12.3)	28	0.0 (.0–12.3)
GMR^c^	altSonflex3M + altSonflex6M	…	…	68	19.6 (13.4–28.6)	67	20.0 (13.6–29.4)	…	…	…	…
	altSonflex3M	…	…	…	…	…	…	29	18.1 (9.0–36.5)	26	22.8 (11.9–43.6)
	altSonflex6M	…	…	…	…	…	…	33	11.4 (6.6–19.9)	33	20.1 (12.2–33.3)
	Placebo	34	…	32	0.9 (.8–1.1)	32	0.9 (.7–1.1)	28	1.0 (.8–1.2)	28	1.0 (.8–1.3)
SBA											
GMT, IC_50_	altSonflex3M + altSonflex6M	18	21.5 (14.5–31.7)	18	1442.2 (485.6–4283.4)	18	911.2 (345.2–2405.0)	…	…	…	…
	altSonflex3M	…	…	…	…	…	…	8	1151.4 (412.1–3216.7)	7	764.7 (259.7–2252.1)
	altSonflex6M	…	…	…	…	…	…	9	878.4 (527.7–1462.1)	9	589.6 (194.3–1789.3)
	Placebo	10	21.4 (11.9–38.5)	10	22.1 (11.4–42.8)	10	22.9 (12.9–40.5)	10	22.1 (11.4–42.9)	10	23.8 (12.4–45.7)
4-fold increase,^c^%	altSonflex3M + altSonflex6M	…	…	18	83.3 (58.6–96.4)	18	83.3 (58.6–96.4)	…	…	…	…
	altSonflex3M	…	…	…	…	…	…	8	100 (63.1–100)	7	71.4 (29.0–96.3)
	altSonflex6M	…	…	…	…	…	…	9	100 (66.4–100)	9	88.9 (51.8–99.7)
	Placebo	…	…	10	0.0 (.0–30.8)	10	0.0 (.0–30.8)	10	0.0 (.0–30.8)	10	0.0 (.0–30.8)
GMR^c^	altSonflex3M + altSonflex6M	…	…	18	67.1 (23.5–192.1)	18	42.4 (17.3–104.0)	…	…	…	…
	altSonflex3M	…	…	…	…	…	…	8	38.5 (13.5–109.7)	7	23.5 (7.2–76.4)
	altSonflex6M	…	…	…	…	…	…	9	53.2 (32.0–88.6)	9	35.7 (11.8–108.4)
	Placebo	…	…	10	1.0 (1.0–1.1)	10	1.1 (.9–1.3)	10	1.0 (1.0–1.1)	10	1.1 (.9–1.3)

Data are value or percentage (95% confidence interval).

Abbreviations: ELISA, enzyme-linked immunosorbent assay; EU, ELISA unit; GMC, geometric mean concentration; GMR, geometric mean ratio; GMT, geometric mean titer; IC_50_, half-maximal (50%) inhibitory concentration; n, number of participants in the group; SBA, serum bactericidal assay.

^a^Assessment time point for altSonflex3M group.

^b^Assessment time point for altSonflex6M group.

^c^Comparison versus baseline. altSonflex3M + altSonflex6M, pooled data for altSonflex3M and altSonflex6M groups; altSonflex3M, participants randomized to receive altSonflex1-2-3 vaccine at 3-month interval; altSonflex6M, participants randomized to receive altSonflex1-2-3 vaccine at 6-month interval; placebo, participants randomized to receive placebo.

Seroresponse rate (ie, a ≥ 4-fold antibody concentration increase vs baseline) was 77.6% after injection 1 (pooled altSonflex1-2-3 groups) and 84.6% and 78.8% after injection 2 in altSonflex3M and altSonflex6M, respectively (Table 1).

Baseline SBA GMTs were close to the LLOQ (33 IC_50_) in both altSonflex1-2-3 and placebo groups (21.5 IC_50_ vs 21.4 IC_50_, respectively) (Supplementary Material 7). At day 29, GMTs were 911.2 IC_50_ in altSonflex1-2-3 groups versus 22.9 IC_50_ in the placebo group. After injection 2, GMTs slightly decreased in both groups but remained well above the baseline levels. GMRs were 42.4 after injection 1 (pooled altSonflex1-2-3 groups), and 23.5 and 35.7 after injection 2 in altSonflex3M and altSonflex6M, respectively. No SBA activity increase was observed after placebo administration (Table 1 and Figure 2).

SBA seroresponse rate was 83.3% after injection 1 (pooled altSonflex1-2-3 groups), and 71.4% and 88.9% after injection 2 in altSonflex3M and altSonflex6M, respectively (Table 1).

#### Shigella flexneri 2a

**Figure 3.**
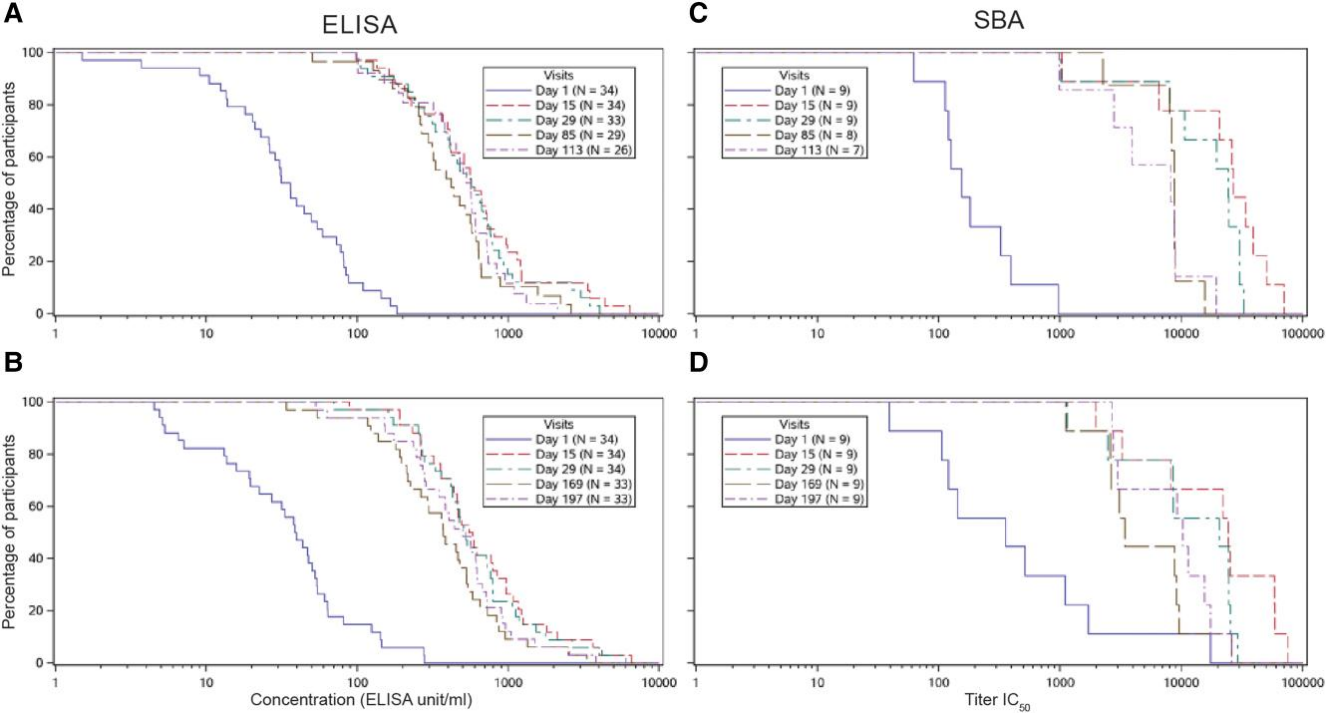
Reverse cumulative distribution curves of (*A* and *B*) anti-*Shigella flexneri* 2a O-antigen serum IgG as measured by ELISA: (*A*) altSonflex3M group up to 1 month after injection 2; (*B*) altSonflex6M group up to 1 month after injection 2; and (*C* and *D*) bactericidal antibody activity as measured by SBA in a subset of participants: (*C*) altSonflex3M group up to 1 month after injection 2; (*D*) altSonflex6M group up to 1 month after injection 2. Abbreviations: altSonflex3M, participants randomized to receive altSonflex1-2-3 vaccine at 3-month interval; altSonflex6M, participants randomized to receive altSonflex1-2-3 vaccine at 6-month interval; ELISA, enzyme-linked immunosorbent assay; EU, ELISA unit; IgG, immunoglobulin G; n, number of participants with available data at a given time point; SBA, serum bactericidal assay.

At baseline, the majority of participants in both altSonflex1-2-3 and the placebo groups had values ≥ LLOQ (3 EU/mL) as measured by ELISA (Supplementary Material 7), with GMCs of 33.0 EU/mL and 44.5 EU/mL, respectively. After injection 1, GMCs were 621.4 EU/mL at day 15 and 564.2 EU/mL at day 29 in altSonflex1-2-3 versus 42.8 EU/mL in placebo groups. Before injection 2, GMCs were 411.8 EU/mL and 364.9 EU/mL in altSonflex3M (day 85) and altSonflex6M (day 169), respectively versus 41.4 EU/mL in the placebo group. After injection 2, GMCs were similar to after injection 1 levels. GMRs were 16.8 after injection 1 (pooled altSonflex1-2-3 groups), and 13.0 and 14.7 after injection 2 in altSonflex3M and altSonflex6M groups, respectively. No increase in responses was observed in the placebo group (Table 2 and Figure 3).

**Table 2.** Immune Response Against *Shigella flexneri* 2a as Measured by ELISA and SBA (Per-Protocol Set)

		Day 1, Baseline		Day 15		Day 29		Day 85^a^/Day 169,^b^ Before Injection 2		Day 113^a^/Day 197^b^	
Parameter	Study Group	n	Value	n	Value or %	n	Value or %	n	Value or %	n	Value or %
ELISA											
GMC, EU/mL	altSonflex3M + altSonflex6M	68	33.0 (25.5–42.7)	68	621.4 (494.3–781.3)	67	564.2 (451.7–704.6)	…	…	…	…
	altSonflex3M	…	…	…	…	…	…	29	411.8 (300.4–564.6)	26	473.6 (351.1–638.8)
	altSonflex6M	…	…	…	…	…	…	33	364.9 (259.9–512.5)	33	451.0 (327.8–620.6)
	Placebo	34	44.5 (27.3–72.6)	32	44.3 (27.4–71.6)	32	42.8 (26.3–69.5)	28	41.4 (23.3–73.6)	28	43.8 (25.1–76.3)
4-fold increase^c^, %	altSonflex3M + altSonflex6M	…	…	68	92.6 (83.7–97.6)	67	91.0 (81.5–96.6)	…	…	…	…
	altSonflex3M	…	…	…	…	…	…	29	93.1 (77.2–99.2)	26	100 (86.8–100)
	altSonflex6M	…	…	…	…	…	…	33	93.9 (79.8–99.3)	33	97.0 (84.2–99.9)
	Placebo	…	…	32	0.0 (.0–10.9)	32	0.0 (.0–10.9)	28	0.0 (.0–12.3)	28	0.0 (.0–12.3)
GMR^c^	altSonflex3M + altSonflex6M	…	…	68	18.8 (14.5–24.5)	67	16.8 (13.1–21.5)	…	…	…	…
	altSonflex3M	…	…	…	…	…	…	29	11.6 (8.2–16.5)	26	13.0 (9.7–17.4)
	altSonflex6M	…	…	…	…	…	…	33	11.9 (8.8–16.2)	33	14.7 (10.9–19.9)
	Placebo	…	…	32	1.0 (.9–1.0)	32	0.9 (.9–1.0)	28	0.8 (.6–1.0)	28	0.9 (.7–1.0)
SBA											
GMT, IC_50_	altSonflex3M + altSonflex6M	18	286.0 (139.8–585.4)	18	18765 (10038–35077)	18	12512 (7195.0–21760)	…	…	…	…
	altSonflex3M	…	…	…	…	…	…	8	7806.8 (4950.2–12312)	7	5397.6 (2193.4–13283)
	altSonflex6M	…	…	…	…	…	…	9	4893.9 (2352.8–10179)	9	8204.6 (4230.5–15912)
	Placebo	10	201.6 (90.6–448.6)	10	295.2 (122.3–712.7)	10	182.4 (75.7–439.7)	10	357.2 (161.9–788.1)	10	451.7 (200.1–1020.0)
4-fold increase^c^, %	altSonflex3M + altSonflex6M	…	…	18	94.4 (72.7–99.9)	18	94.4 (72.7–99.9)	…	…	…	…
	altSonflex3M	…	…	…	…	…	…	8	100 (63.1–100)	7	85.7 (42.1–99.6)
	altSonflex6M	…	…	…	…	…	…	9	88.9 (51.8–99.7)	9	88.9 (51.8–99.7)
	Placebo	…	…	10	0.0 (.0–30.8)	10	0.0 (.0–30.8)	10	0.0 (.0–30.8)	10	10.0 (.3–44.5)
GMR^c^	altSonflex3M + altSonflex6M	…	…	18	65.6 (26.8–160.6)	18	43.7 (18.6–102.6)	…	…	…	…
	altSonflex3M	…	…	…	…	…	…	8	35.0 (17.7–69.5)	7	22.0 (6.1–78.9)
	altSonflex6M	…	…	…	…	…	…	9	11.6 (4.0–33.5)	9	19.4 (5.4–69.1)
	Placebo	…	…	10	1.5 (1.1–2.0)	10	0.9 (.7–1.1)	10	1.8 (1.4–2.2)	10	2.2 (1.5–3.4)

Data are value or percentage (95% confidence interval).

Abbreviations: ELISA, enzyme-linked immunosorbent assay; EU, ELISA unit; GMC, geometric mean concentration; GMR, geometric mean ratio; GMT, geometric mean titer; IC_50_, half-maximal (50%) inhibitory concentration; n, number of participants in the group; SBA, serum bactericidal assay.

^a^Assessment time point for altSonflex3M group.

^b^Assessment time point for altSonflex6M group.

^c^Comparison versus baseline. altSonflex3M + altSonflex6M, pooled data for altSonflex3M and altSonflex6M groups; altSonflex3M, participants randomized to receive altSonflex1-2-3 vaccine at 3-month interval; altSonflex6M, participants randomized to receive altSonflex1-2-3 vaccine at 6-month interval; placebo, participants randomized to receive placebo.

After injection 1, seroresponse rate was 91.0% (pooled altSonflex1-2-3 groups), and after injection 2, 100% (day 113) and 97.0% (day 197), in altSonflex3M and altSonflex6M groups, respectively (Table 2).

Baseline SBA GMTs were 286 IC_50_ versus 201.6 IC_50_ in altSonflex1-2-3 and placebo groups, respectively (Table 2 and Supplementary Material 7). At day 29, GMTs were 12 512 IC_50_ in altSonflex1-2-3 versus 182.4 IC_50_ in the placebo groups. After injection 2, GMTs were similar to after injection 1 levels. GMRs were 43.7 after injection 1 (pooled altSonflex1-2-3 groups), and 22.0 and 19.4 after injection 2 in altSonflex3M and altSonflex6M groups, respectively. No SBA activity increase was observed in the placebo group (Table 2 and Figure 3).

SBA seroresponse rate was 94.4% after injection 1 and 85.7% and 88.9% after injection 2 in altSonflex3M and altSonflex6M groups, respectively (Table 2).

#### Shigella flexneri 1b and S. flexneri 3a

Immune responses against *S. flexneri* 1b and *S. flexneri* 3a showed a similar trend as observed for *S*. *sonnei* and *S. flexneri* 2a, with similar and significantly higher than baseline after injection 1 and after injection 2 GMCs/GMTs, but with quantitatively lower values (Table 3 and Table 4, and Supplementary Material 7). Reverse cumulative distribution ELISA and SBA curves for *S. flexneri* 1b and *S. flexneri* 3a are shown in Supplementary Figures 3 and 4, respectively.

**Table 3.** Immune Response Against *Shigella flexneri* 1b as Measured by ELISA and SBA (Per-Protocol Set)

Parameter	Study Group	Day 1, Baseline		Day 15		Day 29		Day 85^a^/Day 169,^b^ Before Injection 2		Day 113^a^/Day 197^b^	
		n	Value	n	Value or %	n	Value or %	n	Value or %	n	Value or %
ELISA											
GMC, EU/mL	altSonflex3M + altSonflex6M	68	23.2 (16.2–33.2)	68	112.1 (79.1–159.0)	67	103.1 (73.1–145.4)	…	…	…	…
	altSonflex3M	…	…	…	…	…	…	29	79.1 (45.3–138.1)	26	129.7 (79.6–211.5)
	altSonflex6M	…	…	…	…	…	…	33	48.5 (30.1–78.0)	33	73.0 (48.6–109.7)
	Placebo	34	26.9 (15.3–47.3)	32	27.4 (16.1–46.7)	32	27.0 (15.8–46.3)	28	27.2 (15.5–47.7)	28	26.1 (14.6–46.9)
4-fold increase^c^, %	altSonflex3M + altSonflex6M	…	…	68	48.5 (36.2–61.0)	67	43.3 (31.2–56.0)	…	…	…	…
	altSonflex3M	…	…	…	…	…	…	29	24.1 (10.3–43.5)	26	42.3 (23.4–63.1)
	altSonflex6M	…	…	…	…	…	…	33	15.2 (5.1–31.9)	33	30.3 (15.6–48.7)
	Placebo	…	…	32	0.0 (.0–10.9)	32	0.0 (.0–10.9)	28	0.0 (.0–12.3)	28	0.0 (.0–12.3)
GMR^c^	altSonflex3M + altSonflex6M	…	…	68	4.8 (3.6–6.6)	67	4.3 (3.3–5.7)	…	…	…	…
	altSonflex3M	…	…	…	…	…	…	29	3.1 (2.1–4.4)	26	4.7 (2.9–7.8)
	altSonflex6M	…	…	…	…	…	…	33	2.2 (1.7–2.9)	33	3.4 (2.5–4.5)
	Placebo	…	…	32	1.0 (.8–1.2)	32	1.0 (.8–1.2)	28	1.0 (.8–1.1)	28	0.9 (.8–1.1)
SBA											
GMT, IC_50_	altSonflex3M + altSonflex6M	18	8417.6 (3871.6–18302)	18	46293 (30160–71057)	18	56972 (43473–74662)	…	…	…	…
	altSonflex3M	…	…	…	…	…	…	8	58211 (29828–114E3)	7	85066 (42119–172E3)
	altSonflex6M	…	…	…	…	…	…	9	30114 (20412–44427)	9	103E3 (49522–213E3)
	Placebo	10	6573.9 (1649.9–26194)	10	8181.0 (2337.8–28629)	10	8071.2 (2480.5–26262)	10	18084 (8945.6–36558)	10	25229 (12693–50148)
4-fold increase^c^, %	altSonflex3M + altSonflex6M	…	…	18	55.6 (30.8–78.5)	18	55.6 (30.8–78.5)	…	…	…	…
	altSonflex3M	…	…	…	…	…	…	8	37.5 (8.5–75.5)	7	57.1 (18.4–90.1)
	altSonflex6M	…	…	…	…	…	…	9	22.2 (2.8–60.0)	9	66.7 (29.9–92.5)
	Placebo	…	…	10	10.0 (.3–44.5)	10	0.0 (.0–30.8)	10	30.0 (6.7–65.2)	10	40.0 (12.2–73.8)
GMR^c^	altSonflex3M + altSonflex6M	…	…	18	5.5 (2.3–13.2)	18	6.8 (3.1–14.7)	…	…	…	…
	altSonflex3M	…	…	…	…	…	…	8	6.1 (1.7–22.2)	7	9.2 (1.3–63.3)
	altSonflex6M	…	…	…	…	…	…	9	3.2 (1.1–8.9)	9	10.8 (2.9–40.6)
	Placebo	…	…	10	1.2 (.7–2.3)	10	1.2 (.8–2.0)	10	2.8 (1.1–6.8)	10	3.8 (1.4–10.7)

Data are value or percentage (95% confidence interval).

Abbreviations: ELISA, enzyme-linked immunosorbent assay; EU, ELISA unit; GMC, geometric mean concentration; GMR, geometric mean ratio; GMT, geometric mean titer; IC_50_, half-maximal (50%) inhibitory concentration; n, number of participants in the group; SBA, serum bactericidal assay.

^a^Assessment time point for altSonflex3M group.

^b^Assessment time point for altSonflex6M group.

^c^Comparison versus baseline. altSonflex3M + altSonflex6M, pooled data for altSonflex3M and altSonflex6M groups. altSonflex3M, participants randomized to receive altSonflex1-2-3 vaccine at 3-month interval; altSonflex6M, participants randomized to receive altSonflex1-2-3 vaccine at 6-month interval; placebo, participants randomized to receive placebo.

**Table 4.** Immune Response Against *Shigella flexneri* 3a as Measured by ELISA and SBA (Per-Protocol Set)

		Day 1, Baseline		Day 15		Day 29		Day 85^a^/Day 169,^b^ Before Injection 2		Day 113^a^/Day 197^b^	
Parameter	Study Group	n	Value	n	Value or %	n	Value or %	n	Value or %	n	Value or %
ELISA											
GMC, EU/mL	altSonflex3M + altSonflex6M	68	15.6 (11.2–21.9)	68	61.4 (44.1–85.5)	67	58.6 (41.9–82.2)	…	…	…	…
	altSonflex3M	…	…	…	…	…	…	29	26.9 (16.7–43.4)	26	43.1 (26.9–69.2)
	altSonflex6M	…	…	…	…	…	…	33	24.7 (14.1–43.2)	33	37.2 (22.4–61.7)
	Placebo	34	19.9 (13.1–30.2)	32	18.4 (12.2–27.8)	32	19.3 (12.7–29.4)	28	10.1 (6.4–15.9)	28	9.2 (5.6–15.0)
4-fold increase^c^, %	altSonflex3M + altSonflex6M	…	…	68	42.6 (30.7–55.2)	67	43.3 (31.2–56.0)	…	…	…	…
	altSonflex3M	…	…	…	…	…	…	29	6.9 (.8–22.8)	26	23.1 (9.0–43.6)
	altSonflex6M	…	…	…	…	…	…	33	12.1 (3.4–28.2)	33	27.3 (13.3–45.5)
	Placebo	…	…	32	0.0 (.0–10.9)	32	0.0 (.0–10.9)	28	0.0 (.0–12.3)	28	0.0 (.0–12.3)
GMR^c^	altSonflex3M + altSonflex6M	…	…	68	3.9 (3.1–4.9)	67	3.6 (2.9–4.4)	…	…	…	…
	altSonflex3M	…	…	…	…	…	…	29	1.5 (1.2–2.0)	26	2.7 (1.9–3.7)
	altSonflex6M	…	…	…	…	…	…	33	1.8 (1.4–2.4)	33	2.7 (2.0–3.6)
	Placebo	…	…	32	0.9 (.7–1.1)	32	0.9 (.9–1.0)	28	0.5 (.4–.6)	28	0.4 (.4–.5)
SBA											
GMT, IC_50_	altSonflex3M + altSonflex6M	18	3583.6 (2188.2–5868.8)	18	13522 (7570.4–24154)	18	12593 (7401.6–21427)	…	…	…	…
	altSonflex3M	…	…	…	…	…	…	8	9481.4 (5525.4–16270)	7	9100.8 (4250.7–19485)
	altSonflex6M	…	…	…	…	…	…	9	9013.8 (5652.3–14374)	9	13046 (7411.1–22965)
	Placebo	10	3162.0 (975.0–10255)	10	3289.1 (1259.9–8587.0)	10	3573.6 (1356.0–9417.7)	10	4367.1 (2222.4–8581.3)	10	4257.0 (2038.0–8891.9)
4-fold increase^c^, %	altSonflex3M + altSonflex6M	…	…	18	55.6 (30.8–78.5)	18	44.4 (21.5–69.2)	…	…	…	…
	altSonflex3M	…	…	…	…	…	…	8	37.5 (8.5–75.5)	7	28.6 (3.7–71.0)
	altSonflex6M	…	…	…	…	…	…	9	33.3 (7.5–70.1)	9	44.4 (13.7–78.8)
	Placebo	…	…	10	10.0 (.3–44.5)	10	10.0 (.3–44.5)	10	10.0 (.3–44.5)	10	10.0 (.3–44.5)
GMR^c^	altSonflex3M + altSonflex6M	…	…	18	3.8 (2.1–6.8)	18	3.5 (2.0–6.1)	…	…	…	…
	altSonflex3M	…	…	…	…	…	…	8	2.5 (1.2–5.0)	7	2.6 (1.0–6.6)
	altSonflex6M	…	…	…	…	…	…	9	2.4 (1.2–4.9)	9	3.5 (1.4–8.6)
	Placebo	…	…	10	1.0 (.6–1.7)	10	1.1 (.7–1.9)	10	1.4 (.8–2.5)	10	1.3 (.8–2.4)

Data are value or percentage (95% confidence interval).

Abbreviations: ELISA, enzyme-linked immunosorbent assay; EU, ELISA unit; GMC, geometric mean concentration; GMR, geometric mean ratio; GMT, geometric mean titer; IC_50_, half-maximal (50%) inhibitory concentration; n, number of participants in the group; SBA, serum bactericidal assay.

^a^Assessment time point for altSonflex3M group.

^b^Assessment time point for altSonflex6M group.

^c^Comparison versus baseline. altSonflex3M + altSonflex6M, pooled data for altSonflex3M and altSonflex6M groups. altSonflex3M, participants randomized to receive altSonflex1-2-3 vaccine at 3-month interval; altSonflex6M, participants randomized to receive altSonflex1-2-3 vaccine at 6-month interval; placebo, participants randomized to receive placebo.

## DISCUSSION

Although there are antimicrobial drugs for treatment of shigellosis, rising resistance among *Shigella* strains necessitates new prophylactic approaches [30, 31]. Epidemiological data and previous results obtained for candidate vaccines support the rationale to develop an OAg-based vaccine.

For all its promise, GMMA technology is still relatively young. GMMA-based vaccines are known to stimulate the innate immune system, thus safety and reactogenicity assessments are paramount [15]. A first-generation GMMA-based 1790GAHB vaccine against *S. sonnei* demonstrated an acceptable safety profile in 5 clinical trials [20, 21, 23, 24]. Additionally, a recent meta-analysis of pooled data from these trials affirmed the vaccine's safety profile, despite the difference in the amount of OAg/protein across individual trials, and limited sample size [32].

The reactogenicity and safety of altSonflex1-2-3 assessed in the present study confirm the safety profile of the 1790GAHB vaccine. No SAEs considered causally related to the vaccine occurred in the study. While solicited administration-site events occurred frequently in altSonflex1-2-3 recipients, few grade 3 AEs were reported. Pain was the predominant administration site event reported among vaccinated participants. Reports of redness and swelling, although more numerous than in the placebo group, were less frequent than pain and of limited severity, similar to that previously reported for 1790GAHB [32]. Overall, the incidence of fever was low across groups. The proportion of participants reporting unsolicited AEs was relatively high across groups (77.9% in altSonflex1-2-3 vs 88.2% in placebo groups), however it was lower when considering AEs related to study intervention (47.1% and 20.6%). In the altSonflex1-2-3 group, overall, events contributing the most to the count of unsolicited AEs reported (related/not related) were “general disorders and administration site conditions” (42.6%), including mostly injection site reactions and general symptoms such as influenza-like illness and fatigue. Occurrence of grade 3 unsolicited AEs was limited (7.4% in altSonflex1-2-3 vs 5.9% in the placebo groups). Frequency of unsolicited AEs could also be due to the selected solicited events collection strategy, because only fever was measured as a solicited systemic event, while other expected signs and symptoms that may occur after any vaccination (eg, fatigue, headache) were collected as unsolicited AEs. The most commonly reported unsolicited AE in this study (headache) was considered as a solicited systemic event in the 1790GAHB studies and other studies [20, 21, 23, 24]. Overall, no safety signals or concerns precluding further vaccine development were identified.

Considering the available data from the predecessor 1790GAHB vaccine, the selected safety follow-up in this study was relatively short (1 month after injection 2, but included collection of poststudy SAEs related to the study intervention) in line with regulatory authority approval.

Following 1 injection of altSonflex1-2-3, a marked increase in both GMCs (ELISA) and GMTs (SBA) was observed for all antiserotype antibodies at day 15 postvaccination. These remained relatively stable from day 15 to 1 month after injection 1 (day 29), with the most pronounced increase observed for *S. sonnei* and *S. flexneri* 2a. This sharp and steep increase could indicate the presence of preexisting immunity (priming) by *Shigella* [23, 24] or an antigenically related pathogen [33, 34]. This is similar to the strong responses also observed after 1 injection of other outer membrane vesicle vaccines, such as *Hemophilus influenzae* type b polyribosylribitol phosphate-*Neisseria meningitidis* outer membrane protein complex vaccine [35]. Immunogenicity data obtained at 3 and 6 months after injection 1 suggest that immune responses can persist for at least 6 months after the first injection, although with a more pronounced GMC decrease for *S. sonnei* and *S. flexneri* 1b, suggestive of serotype-specific antibody waning. Nevertheless, our results, namely a strong immunologic response following the first injection and a less pronounced recall response following the second injection, are comparable to those obtained for the 1790GAHB vaccine despite the different intervals between administrations (ie, 3 and 6 months in this study vs 1 month in the previous studies) [20, 21, 24]. Similarly, for other OAg-based *Shigella* vaccines, no increase of the response was observed in adults after a second injection at a 4–6-week interval [23, 36, 37]. On the other hand, when a booster injection of the 1790GAHB vaccine was given to adults 3 years after primary vaccination, a strong anamnestic response was observed, showing ability of the GMMA-based vaccine to provide immunological memory [21]. A single dose will significantly affect the eventual uptake of the vaccine and drastically reduce the cost of vaccination. In this study, the vaccine was tested in healthy European adults, who are very different from the target population for vaccination (ie, infants in LMIC countries). In stage 2 of the study, we will test 2 vaccination schedules in infants in Africa to make a final decision regarding the immunization schedule for the target population.

Concerning *anti-S. sonnei* IgG fold increase from baseline, we observed higher GMRs than in previous studies, that is, 20 (95% CI, 13.6–29.4) for altSonflex1-2-3 groups compared with 5.2 (95% CI, 3.5–7.6) at 1.5-µg OAg dose [24] and 4.4 (95% CI, 2.9–6.7) at 6-µg dose [23], and comparable GMRs in another study, that is, 23 (95% CI, 4.9–106) at 6-µg dose [20] for 1790GAHB at 1 month after injection 1. The SBA results suggest a stronger antibody functionality, with GMRs of 42.4 (95% CI, 17.3–104.0) for altSonflex1-2-3 groups compared to 2.5 for 1790GAHB [24] at 1.5-µg OAg dose, to 3.8 [22] and 6.3 at 6-µg dose [19].

Seroresponses against *S. sonnei* and *S. flexneri* 2a, for ELISA or SBA, were robust and relatively stable over time. Although seroresponses for *S. flexneri* 1b and *S. flexneri* 3a were less robust and showed decreasing trends over time, the response patterns were similar for all serotypes.

Lower responses to *S. flexneri* 1b and *S. flexneri* 3a GMMA are difficult to explain based on their similarity in OAg structure and GMMA characteristics to the *S. flexneri* 2a component [25]. Although no negative immune interference was observed in preclinical studies by comparing the 4-component altSonflex1-2-3 formulation to corresponding monocomponent ones [25], it cannot be excluded in humans. Reduced response to polysaccharides sharing a common carrier protein and administered simultaneously has been already reported [38]. When infants received a tetravalent pneumococcal vaccine conjugated to tetanus toxoid (TT; PncT) and a diphtheria-tetanus-pertussis-poliovirus-*H. influenzae* type b (Hib)-TT conjugate vaccine, anti-Hib antibody concentrations were inversely related to TT content. More data will come from stage 2 of the current trial, in which different doses of altSonflex1-2-3 will be tested. Nevertheless, because an accepted correlate of protection is lacking, the relevance of the obtained results to protection from the different strains remains limited.

We observed very high SBA titers at baseline for *S. flexneri* 1b and *S. flexneri* 3a, which was unexpected in this study population (European adults), likely due to the sensitivity of the strains used in the SBA assay. Currently, work is ongoing to identify new strains and optimize the assay for analysis of future trial sera.

There are several strengths of this study. First, the staged study design allowed for initial testing of the vaccine's safety and immunogenicity in European adults, a low-risk and low-vulnerability population with high access to health care, before moving forward to testing in Africa, where shigellosis is endemic, and infants and children are particularly vulnerable to develop this disease. Second, 2 different vaccination schedules, with a shorter (3-month) and a longer (6-month) interval between the injections, allowed assessment of their impact on immunogenicity and will be further tested in stage 2. Additionally, *Shigella* serotypes for the altSonflex1-2-3 vaccine were selected to ensure a broad coverage of the vaccine against epidemiologically relevant *Shigella* strains and based on immune cross-reactivity between *S. flexneri* serotypes evaluated at preclinical and clinical level [7, 25].

The limitations of this study include small group sizes, particularly for the SBA assessments; furthermore, given the ethnicity of the trial population (all participants were white) caution should be exerted when extrapolating these results to other populations, such as those that will be assessed in stage 2 (African and younger population). In addition, a short study follow-up (1 month after injection 2) did not allow for assessment of the persistence of immune responses.

Despite these limitations, to our knowledge, this is the first paper reporting clinical results obtained with a 4-component vaccine against the most relevant *Shigella* serotypes. This is particularly important considering the constant fluctuation of distributions and incidence of *Shigella* serotypes in different locales. Furthermore, the use of the GMMA approach could allow easy introduction of additional serotypes in the vaccine composition as needed.

In conclusion, a single injection of altSonflex1-2-3 generated a strong immune response towards all 4 vaccine *Shigella* serotypes, with the most robust responses against *S. sonnei* and *S. flexneri* 2a, which are the most prevalent serotypes globally. Vaccination schedules tested in stage 1 displayed favorable benefit/risk profiles. Altogether, these results warrant progression to stage 2 of the study, primarily aiming to identify the preferred antigen dose in infants in *Shigella*-endemic regions using an age–de-escalation approach.

## Supplementary Data

Supplementary materials are available at *The Journal of Infectious Diseases* online (http://jid.oxfordjournals.org/). Supplementary materials consist of data provided by the author that are published to benefit the reader. The posted materials are not copyedited. The contents of all supplementary data are the sole responsibility of the authors. Questions or messages regarding errors should be addressed to the author.

## Supplementary Material

jiae273_Supplementary_Data

## Notes

***Acknowledgments.*** The authors thank the study participants, the site study staff, and the GSK study staff involved in the study. The authors thank Giulia Ranzato, Kishor Mariyala, Sateesh Aravapalli, Stefania Barbucci, and Rob Mulder for providing statistical programming activities; and the Akkodis Belgium platform for writing and editorial assistance, manuscript coordination, and design support, on behalf of GSK. Urszula Miecielica provided medical writing support. Alhydrogel is a registered trademark of Croda International.

The *Shigella* project team included the following members: Francesco Citiulo, Emilia Cappelletti, Gianmarco Gasperini, Carlo Giannelli, Alessandra Acquaviva, Luigi Sollai, Renzo Alfini, Maria Grazia Aruta (GSK Vaccines Institute for Global Health Department, GSK, Siena, Italy); and Laura Bartle Martin (US Pharmacopeial Convention, Rockville, MD).

***Author contributions.*** I. L. R., U. N. N., E. S., G. L. C., A. S. B., C. G. V., P. F., E. M., F. N., R. R., I. D. R., J. A., A. M. C., F. B. S., O. R., C. V., F Mi., A. K. A., and A. P. were involved in the conception or the design of the study. I. L. R., U. N. N., C. M., F. Ma., B. J., E. S., A. A., J. J., S. G., G. L. C., A. S. B., C. G. V., P. F., E. M., I. D. R., J. A., A. M. C., F. B. S., O. R., V. C., and A. K. A. participated in the collection or generation of the study/project data. I. L. R., U. N. N., C. M., F. Ma., B. J., E. S., A. A., J. J., P. F., E. M., O. R., V. C., F. Mi., and A. K. A. performed the study/project. U. N. N., F. Ma., E. S., S. G., F. N., A. M. C., O. R., V. C., and F. Mi. contributed to the study with materials/analysis/reagent tools. I. L. R., U. N. N., F. Ma., E. S., A. A., G. L. C., A. S. B., C. G. V., P. F., F. N., R. R., I. D. R., A. M. C., F. B. S., O. R., V. C., F. Mi., A. K. A., and A. P. were involved in the analyses or interpretation of the data. All authors critically revised the manuscript for important intellectual content and approved the version to be published.

***Data availability*.** Data are currently not available as the study remains blinded. Once the study will be unblinded, please refer to GSK weblink to access GSK's data sharing policies and as applicable seek anonymized subject level data via the link https://www.gsk-studyregister.com/en/.

***Trademark statement*.** Alhydrogel is a registered trademark of Croda International.

***Financial support*.** This work was supported by GlaxoSmithKline Biologicals SA, which was involved in all stages of the study conduct and analysis and paid for all costs associated with the development and publishing of this manuscript. A part of the work was supported by the Bill and Melinda Gates Foundation (grant number INV-006482 to F. Mi.).
