# Correction to: Safety and Immunogenicity of a 4-Component Generalized Modules for Membrane Antigens *Shigella* Vaccine in Healthy European Adults: Randomized, Phase 1/2 Study

**DOI:** 10.1093/infdis/jiae564

**Published:** 2024-11-20

In the original publication of this article [Leroux-Roels I, Maes C, Mancini F et al. Safety and Immunogenicity of a 4-Component Generalized Modules for Membrane Antigens *Shigella* Vaccine in Healthy European Adults: Randomized, Phase 1/2 Study. *The Journal of Infectious Diseases,* Volume 230 Issue 4, October 2024, Pages e971-e984, jiae273, https://doi.org/10.1093/infdis/jiae273] an outdated version of Figure 1 was published and has been replaced in the version currently available online. Data and results were unaffected. An erroneous duplicate value of 13.1 in Table 2 has been removed from Day 29 in the row: Parameter GMR with Study Group altSonflex3M + altSonflex6M.

