# Supplementary material for: Safety and Immunogenicity of a 4-Component Generalized Modules for Membrane Antigens Shigella Vaccine in Healthy European Adults: Randomized, Phase 1/2 Study
Source: J Infect Dis. 2024 Jun 10;230(4):e971–84. doi: 10.1093/infdis/jiae273 (PMC11481318; doi:10.1093/infdis/jiae273)
Supplement: jiae273_Supplementary_Data [file jiae273_supplementary_data.zip › GVGH_H06_01TP+Stage1+ms_supplement_for+JID_for+submission.docx]

**Supplement S1.** Inclusion and exclusion criteria

**Inclusion criteria**

Participants eligible for the study were those who at study entry:

- were, in the opinion of the investigator, able to comply with the requirements of the protocol
- provided written or witnessed/thumb printed informed consent prior to performance of any study-specific procedure
- were healthy as established by medical history, clinical examination, and laboratory assessment
- fulfilled all screening requirements
- were seronegative for hepatitis B and C, human immunodeficiency virus, and human leukocyte antigen B27
- were male or female between, and including, 18 and 50 years of age at the time of the first study intervention administration
- female participants of non-childbearing potential were eligible if they had practiced adequate contraception for 1 month prior to study intervention administration, and had a negative pregnancy test on the day of study intervention administration, and agreed to continue adequate contraception during the entire treatment period and for 1 month after completion of the study intervention administration series.

**Exclusion criteria**

Participants not eligible for the study were those who at study entry:

- had a known exposure to *Shigella* at any time prior to study initiation as confirmed during interview with the participant or documented by patient records (e.g., history of microbiologically confirmed *Shigella* infection), recent travel (within 2 years) to a country where *Shigella* or other enteric infections are endemic, or recent occupation (within 3 years) involving *Shigella* species
- had progressive, unstable, or uncontrolled clinical conditions
- had a known or suspected history of any reaction or hypersensitivity likely to be exacerbated by any component of the study vaccine
- had any confirmed or suspected immunosuppressive or immunodeficient condition based on medical history and physical examination (no laboratory testing was required)
- had hypersensitivity, including allergy, to medicinal products or medical equipment whose use was foreseen in this study
- had clinical conditions representing a contraindication to intramuscular vaccination and blood draws
- displayed any behavioral or cognitive impairment or psychiatric disease that, in the opinion of the investigator, could interfere with the participant’s ability to participate in the study
- had an acute disease and/or fever (defined as temperature ≥38.0°C) at the time of enrolment (the participant could still be enrolled into the study at a time when the acute disease and/or fever has resolved)
- had any clinically significant (based on the investigator’s own judgement) hematological and/or biochemical laboratory abnormality
- had a confirmed positive COVID-19 test during the period starting 30 days before the first administration of study vaccines (Day [D] -30 to D1)
- had an acute or chronic illness, clinically significant pulmonary, cardiovascular, hepatic, or renal functional abnormality, as determined by physical examination or laboratory screening tests (participants with a minor illness such as mild diarrhea or mild upper respiratory infection without fever could be enrolled at the discretion of the investigator)
- had any other clinical condition that, in the opinion of the investigator, could pose additional risk to the participant due to participation in the study
- were using long-acting immune-modifying drugs (e.g., infliximab) at any time during the study
- had previously received an experimental *Shigella* vaccine or live *Shigella* challenge
- had received any investigational or non-registered product (drug, vaccine, or medical device) other than the study vaccine during the period starting 30 days before the first injection of study intervention (D -30 to D1), or planned to receive such a product during the study period (use of herbs and traditional treatments was not considered an exclusion criterion)
- had received a vaccine not foreseen by the study protocol during the period starting at -21 days before the first injection (-28 days in the case of live vaccines) and ending after the last injection of study intervention administration. Vaccines allowed by the protocol included flu vaccines and COVID-19 vaccines. The recommended time intervals for administration of these vaccines were at least 7 days before or 7 days after (at least 14 days before or 14 days after in case of live vaccines) each injection of study intervention administration. In case these allowed vaccines were administered outside the aforementioned interval during the study, the participant was to continue to receive the subsequent study intervention administration as per planned schedule. In case emergency mass vaccination for an unforeseen public health threat (e.g., a pandemic) was recommended and/or organized by public health authorities outside the routine immunization program, the time period described above could be reduced if necessary for that vaccine, provided it was used according to the local governmental recommendations and that the sponsor was notified accordingly. If regulations allowed, the recommended time intervals for administration of these vaccines was at least 7 days before or 7 days after (at least 14 days before or 14 days after in case of live vaccines) each injection of study intervention administration.
- had received immunoglobulins and/or any blood products or plasma derivatives, or bone marrow transplantation, during the period starting 3 months before the first injection of study vaccine or had a planned administration of such product during the study period
- had a chronic administration (defined as more than 14 days in total) of immunosuppressants or other immune-modifying drugs during the period starting 3 months prior to the first study vaccine administration (e.g., prednisone equivalent ≥20 mg/day); inhaled and topical steroids were allowed
- were concurrently participating in another clinical study, at any time during the study period, in which the participant has been or will be exposed to an investigational or a non-investigational intervention (drug or invasive medical device)
- were any study personnel or immediate dependents, family, or household member
- were pregnant or lactating women or women planning to become pregnant or planning to discontinue contraceptive precautions
- had a history of or current chronic alcohol consumption and/or drug abuse. Chronic alcohol consumption was defined as one or more of the following: a prolonged period of frequent and heavy alcohol use; the inability to control drinking once it has begun; physical dependence manifested by withdrawal symptoms upon quitting using alcohol; tolerance or the need to use increasing amounts of alcohol to achieve the same effects, and a variety of social and/or legal problems arising from alcohol use.

**Supplement S2.** Details of study objectives and endpoints presented in this paper (Stage 1 – adults in Europe)

| **Objectives** | **Endpoints** |
| --- | --- |
| **Primary** | |
| Safety and reactogenicity of altSonflex1-2-3 in all participants | - number/percentage of participants with solicited administration-site AEs during 7 days after each injection - number/percentage of participants with solicited systemic AEs during 7 days after each injection - number/percentage of participants with unsolicited AEs during 28 days after each injection - number/percentage of participants with SAEs during their entire study participation period (from D1 to D113/D197) - number/percentage of participants with deviations from reference values of hematological, renal, and hepatic panel test results, at 7 days after each injection (D8 and D92/D176) |
| **Secondary** | |
| Immunogenicity profile of altSonflex1-2-3 in all participants | - anti-serotype-specific (i.e., *Shigella sonnei*, *S. flexneri* 1b*, S. flexneri* 2a*, and S. flexneri* 3a) OAg serum IgG GMCs, as measured by ELISA, before each injection (D1 and D85/D169), 14 days after the first injection (D15) and 28 days after each injection (D29 and D113/D197) |
| Further characterization of the immunogenicity profile of the *S. sonnei* component of altSonflex1-2-3 in all participants | - number/percentage of participants achieving an ELISA level equivalent to ≥1:800^a^ titer against *S. sonnei* LPS, before each injection (D1 and D85/D169), 14 days after first injection (D15) and 28 days after each injection (D29 and D113/D197) - number/percentage of participants achieving an ELISA level equivalent to ≥1:1600^a^ titer against *S. sonnei* LPS, before each injection (D1 and D85/D169), 14 days after first injection (D15) and 28 days after each injection (D29 and D113/D197) |
| Evaluation of seroresponse with altSonflex1-2-3 after each vaccination in all participants | - number/percentage of participants showing at least a 4-fold increase in anti-serotype-specific (i.e., *S. sonnei*, *S. flexneri* 1b, *S. flexneri* 2a, and *S. flexneri* 3a) *Shigella* OAg serum IgG concentrations, as measured by ELISA, at 14 days after first injection (D15) and at 28 days after each injection (D29 and D113/D197), compared to baseline (D1 and D85/D169) |
| **Tertiary^b^** | |
| To evaluate the immune response induced by altSonflex1-2-3 in 25% of participants | - anti-serotype-specific (i.e., *S. sonnei*, *S. flexneri* 1b*, S. flexneri* 2a*,* and *S. flexneri* 3a) serum bactericidal antibody GMTs before each injection (D1 and D85/D169), 14 days after the first injection (D15) and 28 days after each injection (D29 and D113/D197) |
| AEs, adverse events; SAEs, serious adverse events; D, day; LPS, lipopolysaccharide; OAg, O-antigen; IgG, immunoglobulin G; GMCs, geometric mean concentrations; GMTs, geometric mean titers; SBA, serum bactericidal assay  ^a^ The 1:800 threshold (known as “Cohen’s Threshold”) and the double of Cohen’s equivalent threshold (1:1600 titer) have been previously reported to be potentially protective for *Shigella* [1, 2] and were calibrated for the assay used in this study to establish the antibody levels corresponding to the median titers reported in convalescent sera and the double of this.  ^b^ The list of the tertiary objectives is not exhaustive. | |

**Supplement S3.** Details of reactogenicity and safety assessment

**Definitions**

An adverse event (AE) was defined as any untoward medical occurrence (an unfavorable/unintended sign, including an abnormal laboratory finding), symptom, or disease (new or exacerbated) temporally associated with the study intervention. The AE may or may not be considered related to the study intervention.

A serious adverse event (SAE) was defined as any untoward medical occurrence that resulted in death; was life-threatening; required hospitalization or prolongation of existing hospitalization; resulted in disability/incapacity, abnormal pregnancy outcome or a congenital anomaly/birth defect in the offspring of a study participant; or as an important medical event that may not be immediately life-threatening or result in death or hospitalization but may jeopardize the participant’s health status or require medical or surgical intervention to prevent any of these outcomes.

Paper diary cards were used to record solicited AEs. Participants were instructed to contact investigators and report any symptoms they perceived as serious. All AEs, SAEs, or other events of interest (e.g., abnormal laboratory values) were followed up at subsequent visits/participant-investigator contacts until event resolution or loss to follow-up.

**Grading of adverse events/serious adverse events**

Solicited AEs were graded 1–3, as follows:

|  | **Grade 1 (mild)** | **Grade 2 (moderate)** | **Grade 3 (severe)** |
| --- | --- | --- | --- |
| **Pain** | Any pain neither interfering with nor preventing normal everyday activities | Painful when limb is moved and interferes with everyday activities | Significant pain at rest. Prevents normal everyday activities |
| **Redness/swelling (diameter)** | ≥20–≤50 mm | >50–≤100 mm | >100 mm |
| **Fever** | ≥38.0°C–<39.0°C | ≥39.0°C–≤40.0°C | >40.0°C |

For all unsolicited AEs (including SAEs) recorded during the study, intensity grade was assessed by the investigator, based on the following categories:

| **Grade** |  |
| --- | --- |
| **1** | An AE which is easily tolerated by the participant, causing minimal discomfort, and not interfering with everyday activities |
| **2** | An AE which is sufficiently discomforting to interfere with normal everyday activities |
| **3** | An AE which prevents normal, everyday activities, e.g., attendance at work, and would necessitate the administration of corrective therapy |

**Grading scales for hematology and biochemistry safety laboratory assessments in Stage 1**

| **Laboratory test** | **Mild**  **(Grade 1)** | **Moderate**  **(Grade 2)** | **Severe**  **(Grade 3)** | **Potentially life-threatening**  **(Grade 4)** |
| --- | --- | --- | --- | --- |
| Creatinine (female), mg/dL | >0.96–1.7 | >1.7–2.0 | >2.0–2.5 | >2.5 or requires dialysis |
| Creatinine (male), mg/dL | >1.17–1.7 |  |  |  |
| Liver function tests –ALT, AST  increase by factor | ≥1.1–2.5 x  ULN | >2.5–5.0 x  ULN | >5.0–10 x  ULN | >10 x ULN |
| Hemoglobin (female), g/dL | 11.0–<11.8 | 9.5–<11.0 | 8.0–<9.5 | <8.0 |
| Hemoglobin (male), g/dL | 12.5–<12.9 | 10.5–<12.5 | 8.5–<10.5 | <8.5 |
| Hemoglobin (female/male)  change from baseline value, g/dL | Any decrease–  1.5 | >1.5–2.0 | >2.0–5.0 | >5.0 |
| WBC increase, cell/mm^3^ | >9300–15 000 | >15 000–20 000 | >20 000–25 000 | >25 000 |
| WBC decrease, cell/mm^3^ | 2500–<3650 | 1500–<2500 | 1000–<1500 | <1000 |
| Platelets decrease (female), cell/mm^3^ | 125 000–<171 000 | 100 000–<125 000 | 25 000–<100 000 | <25 000 |
| Platelets decrease (male), cell/mm^3^ | 125 000–<149 000 |  |  |  |
| Neutrophils decrease, cell/mm^3^ | 1500–<1573 | 1000–<1500 | 500–<1000 | <500 |
| Lymphocytes decrease, cell/mm^3^ | 750–<1133 | 500–<750 | 250–<500 | <250 |
| Eosinophils, cell/mm^3^ | >273–1500 | >1500–5000 | >5000 | Hypereosinophilic |
| ALT, alanine aminotransferase; AST, aspartate aminotransferase; ULN, upper limit of normal; WBC, white blood cell.  The grading scale has been adapted from the Food and Drug Administration (FDA) Guidance for Industry [3] toxicity grading scale and adjusted considering local reference ranges provided by the site. Parameters not included in the FDA grading scales were not graded; their assessment was based on laboratory reference ranges and medical judgement. | | | | |

**Supplement S4.** Details of immunogenicity assessments

*Shigella* serotype-specific serum anti-O-antigen (OAg) IgG levels were measured by ELISA. *S. sonnei* LPS or *S. flexneri* 1b, *S. flexneri* 2a, *S. flexneri* 3a OAg were used as coating antigens, at final concentrations of 0.5 µg/mL in phosphate-buffered saline (PBS) for *S. sonnei*, 2 µg/mL in carbonate buffer for *S. flexneri* 1b, 0.5 µg/mL in carbonate buffer for *S. flexneri* 2a, 1 µg/mL in PBS for *S. flexneri* 3a, and 1:4500 dilution of goat anti-human IgG alkaline phosphatase secondary antibody (Sigma Aldrich) using the methodology previously described [2, 4, 5]. ELISA units (EU) were expressed relative to a five-parameter human antigen-specific antibody standard serum curve composed of 10 standard points and two blank wells (run in duplicate on each plate). One EU was defined as the reciprocal of the dilution of the standard serum that gives an absorbance value (Optical Density [OD] measured at 405 nm subtracted to OD measured at 490 nm) equal to 1 in this assay. High control and low control (HC and LC, respectively) were run on each plate at appropriate dilution. Standard serum to assess anti-*S. sonnei-*specific antibodies was obtained by pooling sera from four individual high responders initially enrolled in a previous phase IIb clinical trial who received two vaccinations with *S. sonnei* monocomponent 1790GAHB [6], whereas the standard sera used to assess anti-*S. flexneri-*specific antibodies were obtained by pooling sera pre-vaccination from nine individual subjects enrolled in clinical trial H01_02TP conducted in Pakistan and India (ClinicalTrials.gov NCT01229176), where *Shigella* is endemic, who were screened for response against each of the above-mentioned *S. flexneri* serotypes. The anti-*S. sonnei* standard serum was calibrated in order to meet the definition of 1 EU as the reciprocal of the dilution giving an OD equal to 1 for this study (Conti et al. NPJ in press). The primary anti-*S. flexneri* standard sera were calibrated against each coating antigen, and the assays were fit-for-purpose qualified in terms of precision, linearity, specificity and limits of blanks and quantification. Limits of standard curve accuracy were set at the nominal value of the standard at the last and the first serial dilutions, respectively, at which the confidence interval of residual error percentage with 90% probability fell within the acceptance range of [-25%; + 25%].

In each run of analysis, the following quality control criteria were applied: R-square value ≥0.96 for the 5PL curve fit to standard dilution series, maximum background <0.15 OD, minimum value of OD maximum ≥2.6, range between 0.5 OD and 2 OD for 1 EU/mL, standard deviation for the controls with respect to expected value <40%. If at least one of the above-mentioned criteria was not met, the entire layout was repeated under the same experimental conditions. For each sample, the EU/mL were determined as average of EU/mL of the triplicate at each specific sera dilution if the coefficient of variation was <30%, otherwise the sample was re-run under the same conditions.

**Supplement S5.** *Shigella* strains used in the study

The following *Shigella* strains were used to assess functionality of the sera by serum bactericidal activity (SBA): *S. sonnei* 53G virg::cat [7, 8], *S. flexneri* 1b (Stansfield NTCT 5 strain), *S. flexneri* 2a (2457T strain), and *S. flexneri* 3a (6885 strain) [9].

**Supplement S6.** Details of statistical analyses

The following analysis sets were included in the study:

- Enrolled set included all participants who entered the study (who were randomized or received study intervention or underwent a post-screening study procedure)
- Exposed set included all participants who received at least one injection of the study intervention
- Full analysis set included all participants who received at least one injection of the study intervention and had post-vaccination immunogenicity data available
- Per-protocol set (PPS) included all eligible participants who received all injections as per protocol, had post-injection immunogenicity results available, complied with dosing/blood draw intervals, without intercurrent conditions that may have interfered with vaccine immunogenicity and without prohibited concomitant medication/vaccination. The PPS for immunogenicity was defined by timepoint
- Solicited safety set included all participants who received at least one injection of the study intervention and had solicited safety data available
- Unsolicited safety set included all participants who received at least one injection of the study intervention and reported unsolicited AEs or reported not having unsolicited AEs

Solicited AEs were assessed on the solicited safety set, unsolicited AEs were assessed on the unsolicited safety set, and SAEs and hematology and biochemistry safety laboratory values were assessed on the exposed set. Immunogenicity analyses were performed on the PPS.

Percentages of participants who reported solicited AEs, unsolicited AEs, SAEs, deviations in hematological, renal, and hepatic analyses over the different follow-up periods and timepoints were calculated with 95% confidence intervals (95% CIs).

Within-participant geometric mean ratios were computed by taking the antilog of the means of within-participant differences between log-transformed titers after each sampling timepoint and log-transformed titers at baseline/pre-vaccination.

Percentage of participants with ELISA levels equivalent to ≥1:800 (Cohen’s threshold) and ≥1:1600 (double of Cohen’s threshold) equivalent titers against *S. sonnei* LPS were reported with 95% CIs, for each sampling timepoint [1, 10]. Both the 1:800 and 1:1600 thresholds from the Tel Aviv University (TAU) assay were calibrated to the method used in the present study’s analysis and correspond to 161 EU/mL and 396 EU/mL, respectively, in ELISA method used in our study. Of note, for the purpose of this analysis, it was decided to consider as equivalent for each threshold the lower limit of the 95% CI of the point estimate (i.e., 124 EU/mL and 315 EU/mL for ELISA for 800 and 1600 TAU assay titers, respectively). Percentages of participants achieving ELISA IgG antibody levels equivalent to 1:800 and 1:1600 titers are reported in Supplementary Table S3.

The distribution of serotype-specific *Shigella* anti-LPS/OAg serum IgG for study groups at sampling timepoints is displayed graphically using reverse cumulative distribution curves for all serotypes (*S. sonnei*, *S. flexneri* 1b, *S. flexneri* 2a, and *S. flexneri* 3a).

**ELISA and SBA LLOQ cut-off values**

| **Serotype** | **Cut-off value** |
| --- | --- |
| **ELISA** | |
| *Shigella sonnei* | 12.8 EU/mL |
| *S. flexneri* 1b | 6 EU/mL |
| *S. flexneri* 2a | 3 EU/mL |
| *S. flexneri* 3a | 2.9 EU/mL |
| **SBA** | |
| *S. sonnei* | 33 IC_50_ |
| *S. flexneri* 1b | 21.8 IC_50_ |
| *S. flexneri* 2a | 13.4 IC_50_ |
| *S. flexneri* 3a | 82.6 IC_50_ |
| SBA, serum bactericidal assay; LLOQ, lower level of quantification; EU/mL, ELISA unit per mL; IC_50_, serum dilution giving 50% inhibition of bacterial growth | |

**Supplement S7.** Details of immunogenicity results

Percentages of participants achieving ELISA IgG antibody levels equivalent to 1:800 and 1:1600 titers are reported in the Supplementary Table S3 below.

**Baseline geometric mean concentrations (GMCs) as measured by ELISA**

For *S. sonnei*, at baseline, 27/68 participants in the altSonflex1-2-3 groups had baseline values equal to or above the lower limit of quantification (LLOQ; 12.8 EU/mL) vs. 19/34 of participants in the Placebo group. For *S. flexneri* 2a, 67/68 participants in the altSonflex1-2-3 groups and 33/34 of participants in the Placebo group had baseline values ≥LLOQ (3 EU/mL). For *S. flexneri* 1b, 57/68 participants in the altSonflex1-2-3 groups and 27/34 of participants in the Placebo group had baseline values ≥LLOQ (6 EU/mL). For *S. flexneri* 3a, 61/68 participants in the altSonflex1-2-3 groups and 32/34 of participants in the Placebo group had baseline values ≥LLOQ (2.9 EU/mL).

At baseline, anti-*S. flexneri* 1b GMCs were 23.2 EU/mL in the altSonflex groups vs. 26.9EU/mL in the Placebo group, and anti-*S. flexneri* 3a GMCs were 15.6 EU/mL vs. 19.9 EU/mL, respectively.

**Baseline geometric mean titers (GMTs) as measured by SBA**

At baseline, for *S. sonnei*, 2/18 participants in the altSonflex1-2-3 groups had baseline values ≥LLOQ (33 IC_50_) vs. 1/10 of participants in the Placebo group. For *S. flexneri* 2a, 1b and 3a, all participants across the groups had baseline values ≥LLOQ (13.4 IC_50_, 21.8 IC_50_, and 82.6 IC_50_ for *S. flexneri* 2a, 1b, and 3b, respectively).

***S. flexneri* 1b and *S. flexneri* 3a immune responses**

For both *S. flexneri* 1b and *S. flexneri* 3a, GMCs increased post-injection 1 and decreased pre-injection 2 but not below the baseline levels; post-injection 2, anti-*S. flexneri* 1b GMCs reached similar levels as post-injection 1, while anti-*S. flexneri* 3a GMCs were lower than post-injection 1 levels but remained well above the baseline levels.

Post-injection 1, seroresponse rates in both altSonflex1-2-3 groups were 43.3% for both serotypes. Pre-injection 2, seroresponse rates in altSonflex3M and altSonflex6M were 24.1% (D85) and 15.2% (D169) for *S. flexneri* 1b and 6.9% and 12.1%, respectively for *S. flexneri* 3a; post-injection 2, seroresponse rates were 42.3% and 30.3% (*S. flexneri* 1b), and 23.1% and 27.3% (*S. flexneri* 3a), respectively.

Baseline SBA GMTs for both *S. flexneri* 1b and *S. flexneri* 3a were substantially higher than the LLOQ (21.8 IC_50_ and 82.6 IC_50_, respectively) in both altSonflex1-2-3 (*S. flexneri* 1b: 8417.6 IC_50_; *S. flexneri* 3a: 3583.6 IC_50_) and the Placebo group (6573.9 IC_50_; 3162 IC_50_). For both serotypes, a substantial increase in GMTs was observed post-injection 1; pre-injection 2, GMTs remained high at D85 while decreased at D169; post-injection 2, a further increase in GMTs was observed for *S. flexneri* 1b in both altSonflex1-2-3 groups, while for *S. flexneri* 3a, no increase was observed at D113, but GMTs reached similar levels as post-injection 2 in the altSonflex6M group. No SBA activity increase was observed in the Placebo group after vaccination. Post-injection 1, SBA seroresponse rates in the altSonflex1-2-3 groups were 55.6% for *S. flexneri* 1b and 44.4% for *S. flexneri* 3a; pre-injection 2, seroresponse rates were 37.5% and 22.2% (*S. flexneri* 1b) and 37.5% and 33.3% (*S. flexneri* 3a) in altSonflex3M and altSonflex6M groups, respectively, and post-injection 2, 57.1% and 66.7% (*S. flexneri* 1b) and 28.6% and 44.4% (*S. flexneri* 3a), respectively.

**Supplementary Table S1.** Demographic characteristics of study participants (exposed set)

|  | **altSonflex3M** N = 34 | **altSonflex6M** N = 34 | **Placebo** N = 34 | **Total** N = 102 |
| --- | --- | --- | --- | --- |
| Mean age ± SD (years) | 32.4 (9.3) | 34.8 (9.5) | 36.0 (7.2) | 34.4 (8.8) |
| Female, n (%) | 28 (82.4) | 27 (79.4) | 27 (79.4) | 82 (80.4) |
| Race, n (%) |  |  |  |  |
| White | 34 (100) | 34 (100) | 34 (100) | 102 (100) |

N, number of participants; n (%) number (percentage) of participants in a given category; SD, standard deviation.

altSonflex3M, participants randomized to receive altSonflex1-2-3 vaccine at 3-month interval; altSonflex6M, participants randomized to receive altSonflex1-2-3 vaccine at 6-month interval; Placebo, participants randomized to receive placebo.

**Supplementary Table S2.** Demographic characteristics of participants included in the per-protocol set (N=87; Day 113)

| **Parameter** |  | **altSonflex3M** N = 26 | **altSonflex6M** N = 33 | **Placebo** N = 28 | **Total** N = 87 |
| --- | --- | --- | --- | --- | --- |
| Mean age ± SD (years) |  | 32.2 (9.5) | 34.6 (9.6) | 37.1 (6.9) | 34.7 (8.9) |
| Female, n (%) |  | 20 (76.9) | 26 (78.8) | 22 (78.6) | 68 (78.2) |
| Race, n (%) |  |  |  |  |  |
| White |  | 26 (100) | 33 (100) | 28 (100) | 87 (100) |
| N, number of participants; n (%) number (percentage) of participants in a given category; SD, standard deviation.  altSonflex3M, participants randomized to receive altSonflex1-2-3 vaccine at 3-month interval; altSonflex6M, participants randomized to receive altSonflex1-2-3 vaccine at 6-month interval; Placebo, participants randomized to receive placebo. | | | | | |

**Supplementary Table S3.** Percentages of participants achieving anti-*Shigella sonnei* ELISA IgG antibody levels equivalent to 1:800 and 1:1600 titers

|  |  | **D1 (baseline)** | | **D15** | | **D29** | | **D85^a^/D169^b^ (pre-injection 2)** | | **D113^a^/D197^b^** | |
| --- | --- | --- | --- | --- | --- | --- | --- | --- | --- | --- | --- |
| **Parameter** | **Study group** | **N** | **value** | **N** | **value** | **N** | **value** | **N** | **value** | **N** | **value** |
| **≥1:800 titer^c^, % (95% CI)** | altSonflex3M+altSonflex6M | 68 | 1.5 (0.0–7.9) | 68 | 60.3 (47.7–72.0) | 67 | 59.7 (47.0–71.5) |  |  |  |  |
|  | altSonflex3M | - | - | - | - | - | - | 29 | 58.6 (38.9–76.5) | 26 | 80.8 (60.6–93.4) |
|  | altSonflex6M | - | - | - | - | - | - | 33 | 42.4 (25.5–60.8) | 33 | 66.7 (48.2–82.0) |
|  | Placebo | 34 | 14.7 (5.0–31.1) | 32 | 12.5 (3.5–29.0) | 32 | 12.5 (3.5–29.0) | 28 | 10.7 (2.3–28.2) | 28 | 10.7 (2.3–28.2) |
| **≥1:1600 titer^c^, % (95% CI)** | altSonflex3M+altSonflex6M | 68 | 1.5 (0.0–7.9) | 68 | 35.3 (24.1–47.8) | 67 | 34.3 (23.2–46.9) | - | - | - | - |
|  | altSonflex3M | - | - | - | - | - | - | 29 | 41.4 (23.5–61.1) | 26 | 42.3 (23.4–63.1) |
|  | altSonflex6M | -- | - | - | - | - | - | 33 | 27.3 (13.3–45.5) | 33 | 30.3 (15.6–48.7) |
|  | Placebo | 34 | 5.9 (0.7–19.7) | 32 | 6.3 (0.8–20.8) | 32 | 6.3 (0.8–20.8) | 28 | 7.1 (0.9–23.5) | 28 | 7.1 (0.9–23.5) |
| N/%, number/percentage of participants in the group achieving the indicated levels; D, day; CI, confidence interval. ^a^ assessment timepoint for altSonflex3M group. ^b^ assessment timepoint for altSonflex6M group. ^c^ The 1:800 and 1:1600 titers correspond to 124 ELISA units (EU)/mL and 315 EU/mL, respectively, in ELISA used in this study.  altSonflex3M+altSonflex6M, pooled data for altSonflex3M and altSonflex6M groups; altSonflex3M, participants randomized to receive altSonflex1-2-3 vaccine at 3-month interval; altSonflex6M, participants randomized to receive altSonflex1-2-3 vaccine at 6-month interval; Placebo, participants randomized to receive placebo. | | | | | | | | | | | |

**Supplementary Figure S1.** Study design

**
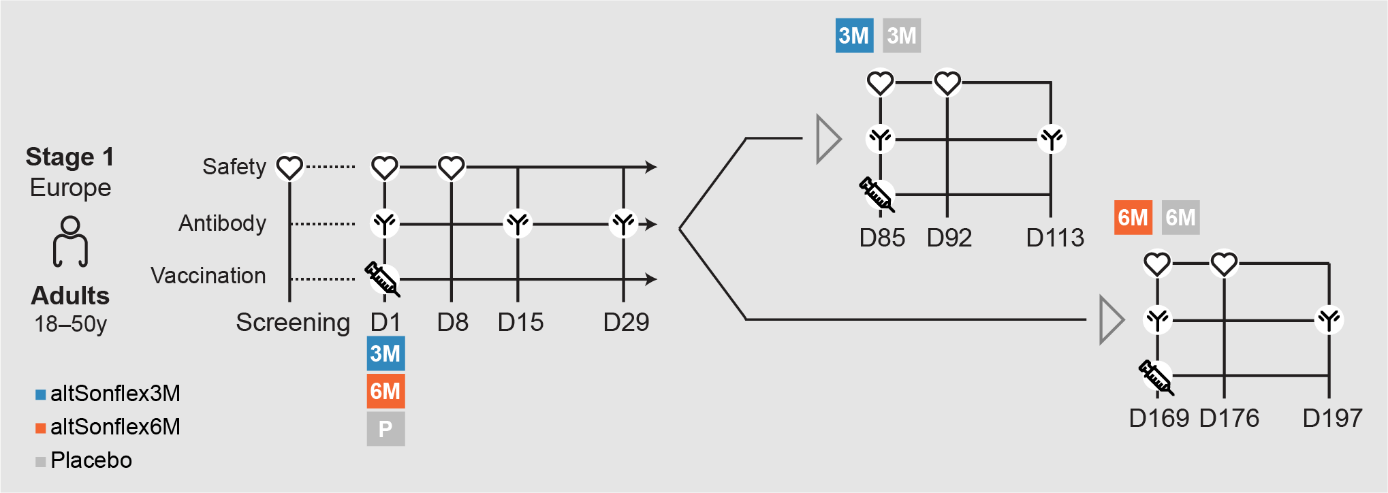
**

**Footnote:** y, years; D, day; M, month; 3M, participants who received study vaccine/placebo at 3-month interval between the 2 injections; 6M, participants who received study vaccine/placebo at 6-month interval between the 2 injections; P, Placebo.

altSonflex3M, participants randomized to receive altSonflex1-2-3 vaccine at 3-month interval; altSonflex6M, participants randomized to receive altSonflex1-2-3 vaccine at 6-month interval; Placebo, participants randomized to receive placebo.

**Supplementary Figure S2.** Participant flow

**
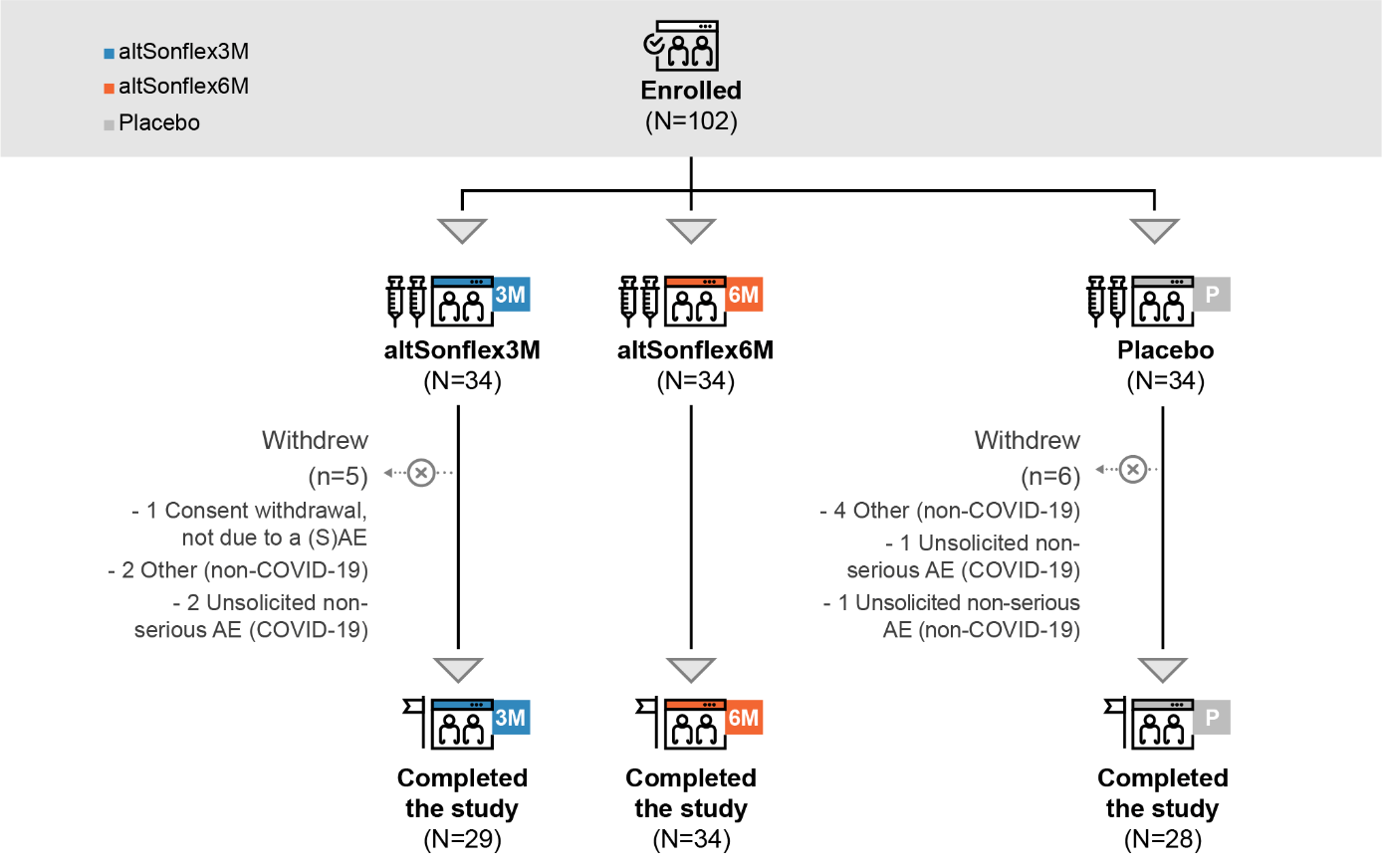
**

**Footnote:** N/n, number of participants; (S)AE, (serious) adverse event; 3M, participants randomized to receive study vaccine/placebo at 3-month interval between the 2 injections; 6M, participants randomized to receive study vaccine/placebo at 6-month interval between the 2 injections; P, Placebo.

altSonflex3M, participants randomized to receive altSonflex1-2-3 vaccine at 3-month interval; altSonflex6M, participants randomized to receive altSonflex1-2-3 vaccine at 6-month interval; Placebo, participants randomized to receive placebo.

**Supplementary Figure S3.** Reverse cumulative distribution curves of anti-*Shigella flexneri* 1b (**A–C**) O-antigen serum IgG as measured by ELISA: **A**. altSonflex3M + altSonflex6M groups (pooled data) post-injection 1; **B**. altSonflex3M group up to 1 month post-injection 2; **C**. altSonflex6M group up to 1 month post-injection 2, and (**D–F**) bactericidal antibody activity, as measured by SBA in a subset of participants: **D**. altSonflex3M and altSonflex6M groups (pooled data) post-injection 1; **E**. altSonflex3M group up to 1 month post-injection 2; **F**. altSonflex6M group up to 1 month post-injection 2


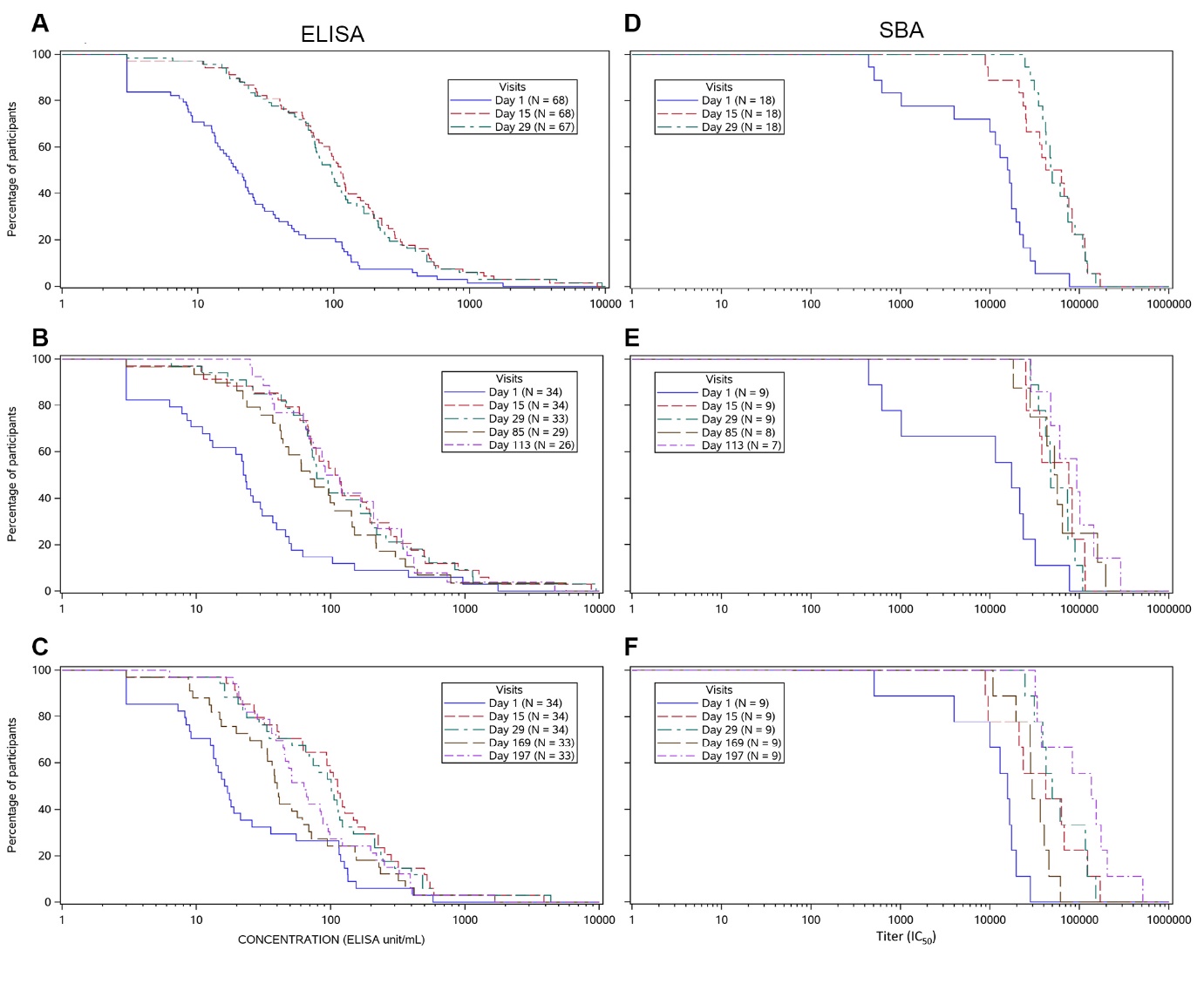


**Footnote:** IgG, immunoglobulin G; SBA, serum bactericidal assay; N, number of participants with available data at a given timepoint. altSonflex3M+altSonflex6M, pooled data for altSonflex3M and altSonflex6M groups; altSonflex3M, participants randomized to receive altSonflex1-2-3 vaccine at 3-month interval; altSonflex6M, participants randomized to receive altSonflex1-2-3 vaccine at 6-month interval.

**Supplementary Figure S4.** Reverse cumulative distribution curves of anti-*Shigella flexneri* 3a (**A–C**) O-antigen serum IgG as measured by ELISA: **A**. altSonflex3M + altSonflex6M groups (pooled data) post-injection 1; **B**. altSonflex3M group up to 1 month post-injection 2; **C**. altSonflex6M group up to 1 month post-injection 2, and (**D–F**) bactericidal antibody activity, as measured by SBA in a subset of participants: **D**. altSonflex3M and altSonflex6M groups (pooled data) post-injection 1; **E**. altSonflex3M group up to 1 month post-injection 2; **F**. altSonflex6M group up to 1 month post-injection 2

**
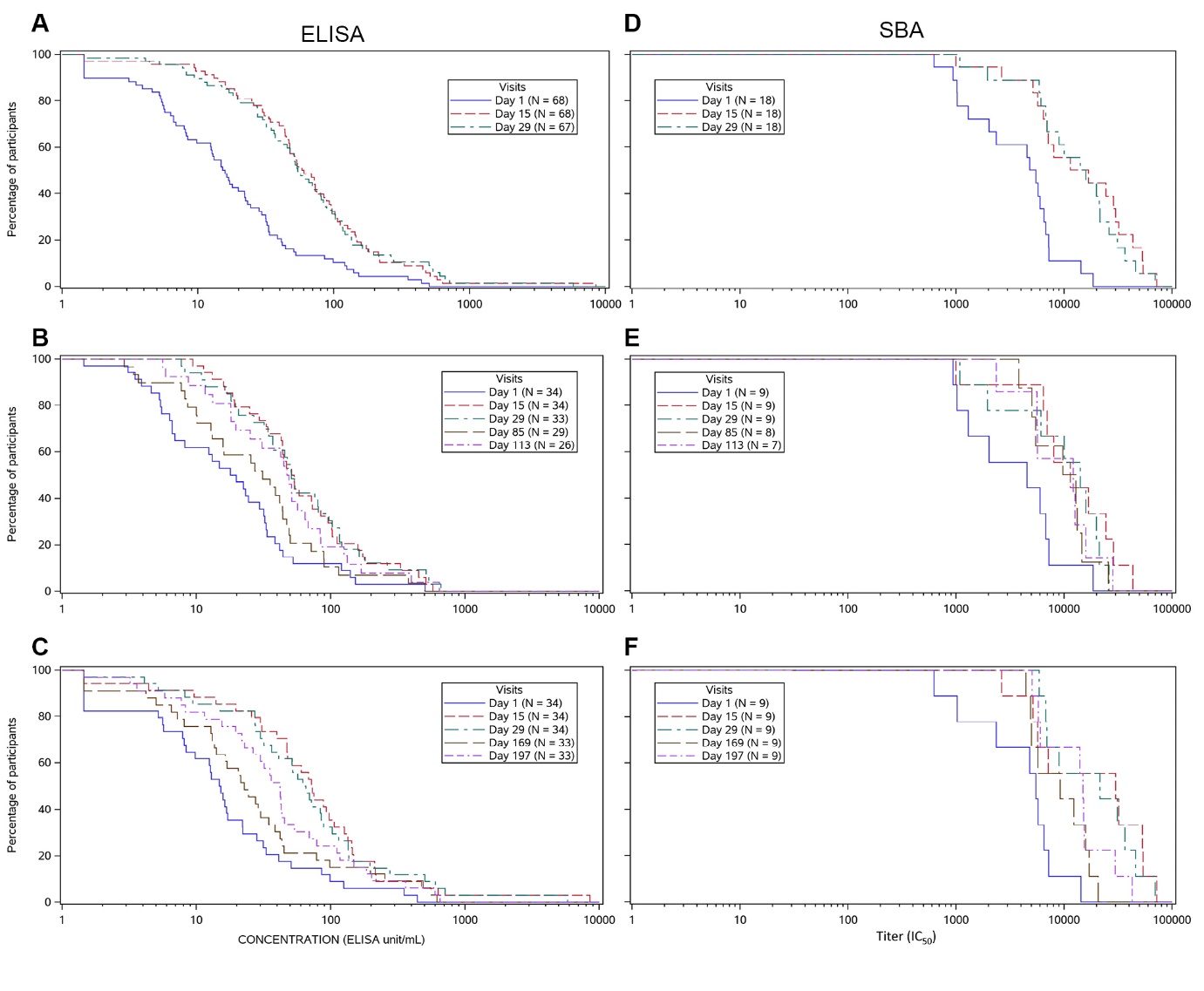
**

**Footnote:** IgG, immunoglobulin G; SBA, serum bactericidal assay; N, number of participants with available data at a given timepoint. altSonflex3M+altSonflex6M, pooled data for altSonflex3M and altSonflex6M groups; altSonflex3M, participants randomized to receive altSonflex1-2-3 vaccine at 3-month interval; altSonflex6M, participants randomized to receive altSonflex1-2-3 vaccine at 6-month interval.

**References**

1. Cohen D, Green MS, Block C, Rouach T, Ofek I. Serum antibodies to lipopolysaccharide and natural immunity to shigellosis in an Israeli military population. J Infect Dis **1988**; 157:1068-71.

2. Launay O, Lewis DJM, Anemona A, et al. Safety Profile and Immunologic Responses of a Novel Vaccine Against Shigella sonnei Administered Intramuscularly, Intradermally and Intranasally: Results From Two Parallel Randomized Phase 1 Clinical Studies in Healthy Adult Volunteers in Europe. EBioMedicine **2017**; 22:164-72.

3. U.S. Food and Drug Administration. Department of Health and Human Services. Center for Biologics Evaluation and Research. Toxicity Grading Scale for Healthy Adult and Adolescent Volunteers Enrolled in Preventive Vaccine Clinical Trials. Guidance for Industry. 2007. Available from: <https://www.fda.gov/regulatory-information/search-fda-guidance-documents/toxicity-grading-scale-healthy-adult-and-adolescent-volunteers-enrolled-preventive-vaccine-clinical>. Accessed on: 9 November 2023.

4. Aruta MG, Lari E, De Simone D, et al. Characterization of Enzyme-Linked Immunosorbent Assay (ELISA) for Quantification of Antibodies against Salmonella Typhimurium and Salmonella Enteritidis O-Antigens in Human Sera. BioTech (Basel) **2023**; 12.

5. Launay O, Ndiaye AGW, Conti V, et al. Booster Vaccination With GVGH Shigella sonnei 1790GAHB GMMA Vaccine Compared to Single Vaccination in Unvaccinated Healthy European Adults: Results From a Phase 1 Clinical Trial. Front Immunol **2019**; 10:335.

6. Frenck RW, Jr., Conti V, Ferruzzi P, et al. Efficacy, safety, and immunogenicity of the Shigella sonnei 1790GAHB GMMA candidate vaccine: Results from a phase 2b randomized, placebo-controlled challenge study in adults. EClinicalMedicine **2021**; 39:101076.

7. Rossi O, Molesti E, Saul A, Giannelli C, Micoli F, Necchi F. Intra-Laboratory Evaluation of Luminescence Based High-Throughput Serum Bactericidal Assay (L-SBA) to Determine Bactericidal Activity of Human Sera against Shigella. High Throughput **2020**; 9:14.

8. Caboni M, Pédron T, Rossi O, et al. An O antigen capsule modulates bacterial pathogenesis in Shigella sonnei. PLoS Pathog **2015**; 11:e1004749.

9. Mancini F, Micoli F, Rossi O. Setup and Characterization of a High-Throughput Luminescence-Based Serum Bactericidal Assay (L-SBA) to Determine Functionality of Human Sera against Shigella flexneri. BioTech (Basel) **2022**; 11:29.

10. Cohen D, Ashkenazi S, Schneerson R, et al. Threshold protective levels of serum IgG to Shigella lipopolysaccharide: re-analysis of Shigella vaccine trials data. Clin Microbiol Infect **2023**; 29:366-71.
