# Supplementary figures and images for: Safety and Immunogenicity of a 4-Component Generalized Modules for Membrane Antigens Shigella Vaccine in Healthy European Adults: Randomized, Phase 1/2 Study
Source: J Infect Dis. 2024 Jun 10;230(4):e971–84. doi: 10.1093/infdis/jiae273 (PMC11481318; doi:10.1093/infdis/jiae273)

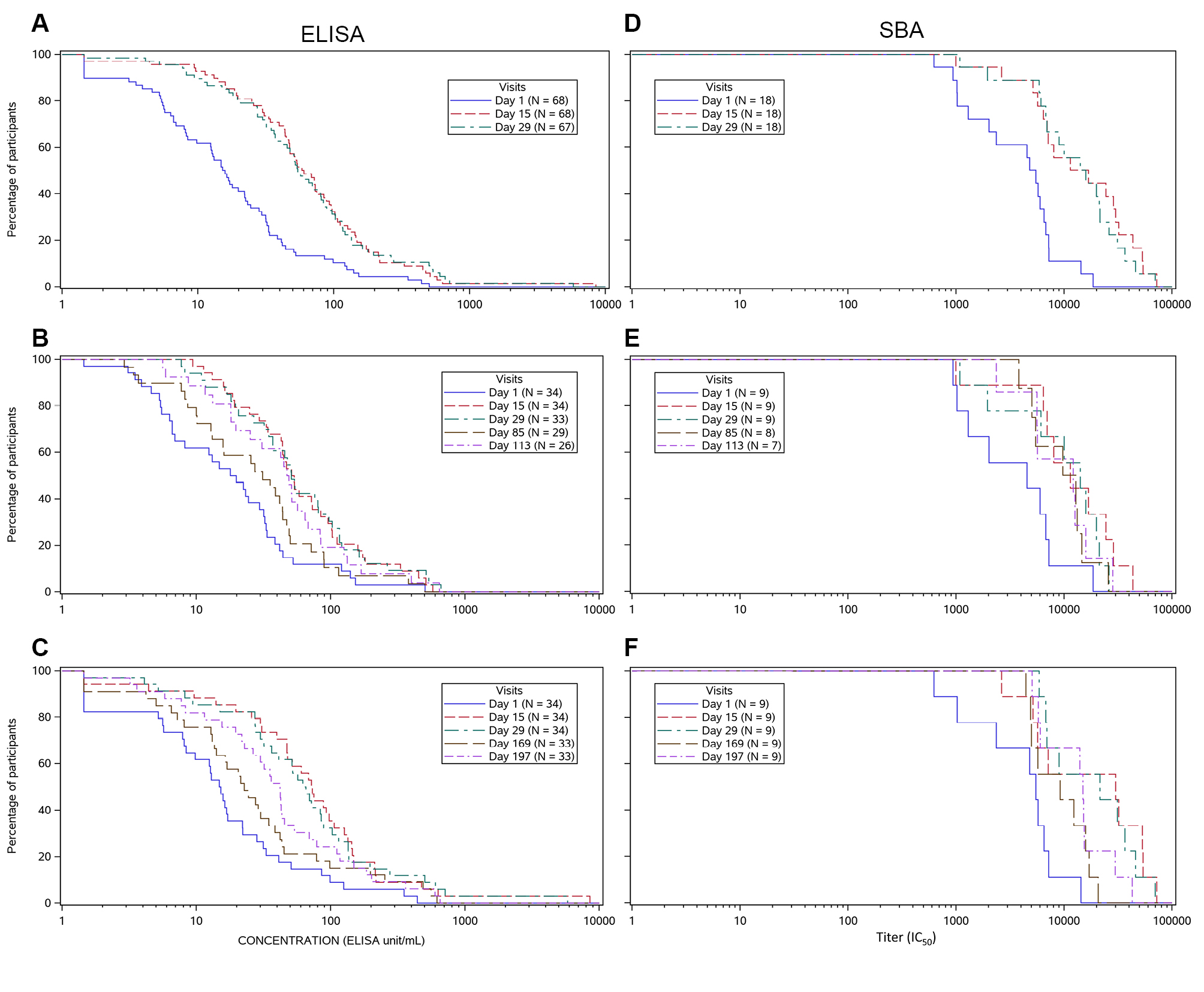

Supplement: jiae273_Supplementary_Data [file jiae273_supplementary_data.zip › SuplFig4_S.flex3a RCD curves.jpg]

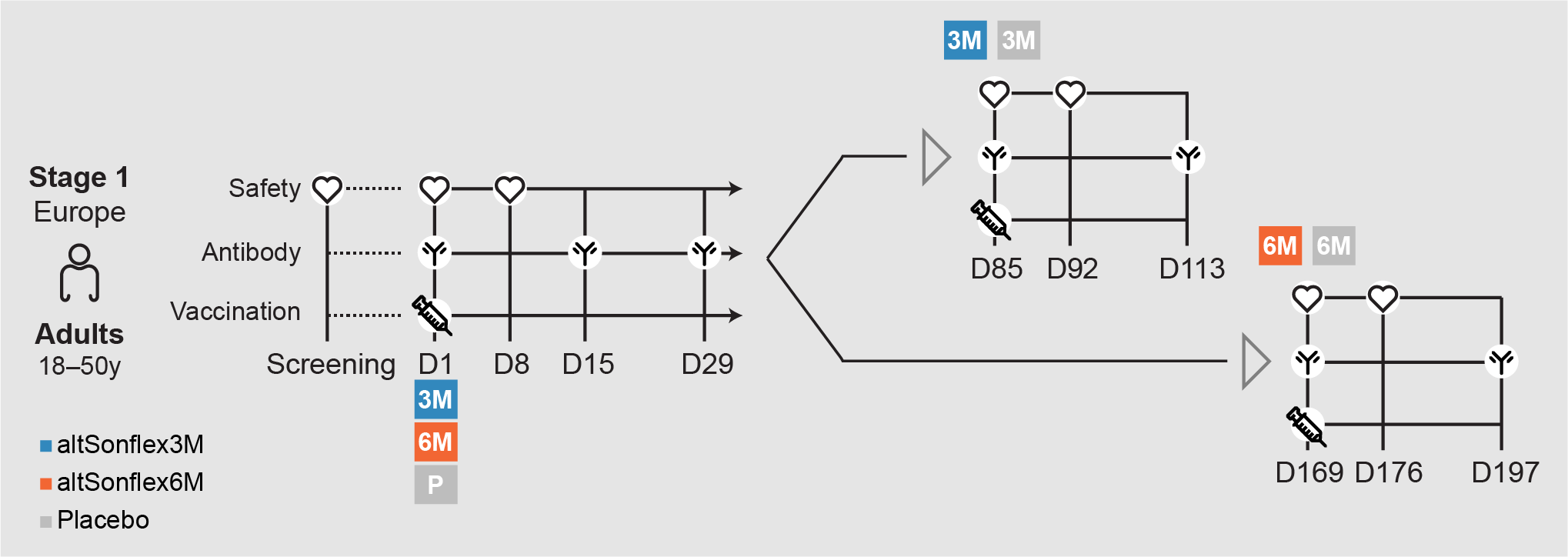

Supplement: jiae273_Supplementary_Data [file jiae273_supplementary_data.zip › SuplFigS1_Study Design.tif]

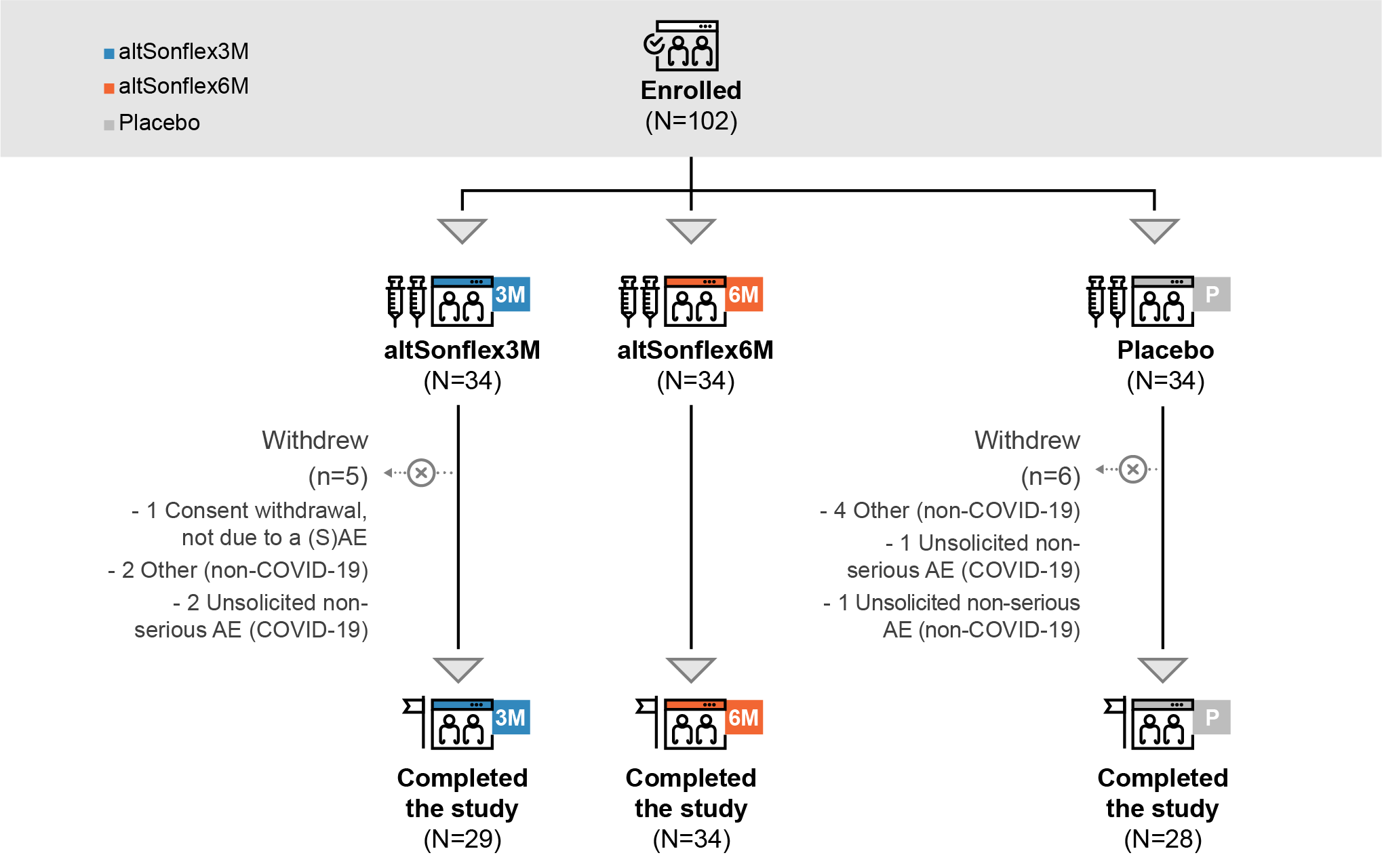

Supplement: jiae273_Supplementary_Data [file jiae273_supplementary_data.zip › SuplFigS2_Participant Flow.tif]

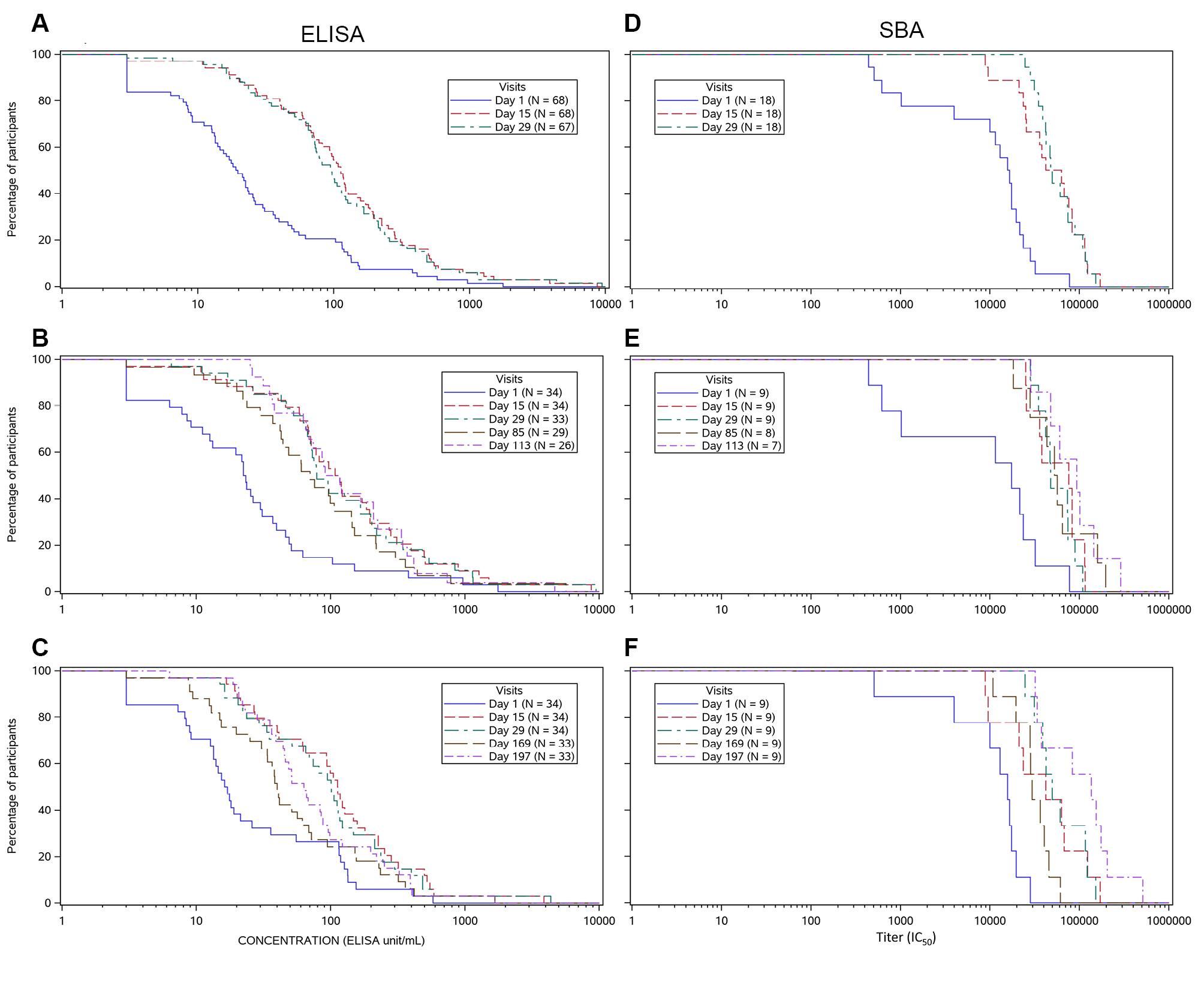

Supplement: jiae273_Supplementary_Data [file jiae273_supplementary_data.zip › SuplFigS3_S.flex1b RCD curves.jpg]
